# A Comparison of Additional Benefit Assessment Methods for Time-to-Event Endpoints Using Hazard Ratio Point Estimates or Confidence Interval Limits by Means of a Simulation Study

**DOI:** 10.1177/0272989X241239928

**Published:** 2024-05-09

**Authors:** Christopher A. Büsch, Marietta Kirchner, Rouven Behnisch, Meinhard Kieser

**Affiliations:** Institute of Medical Biometry (IMBI), Department Medical Biometry, Heidelberg University, Heidelberg, Germany; Institute of Medical Biometry (IMBI), Department Medical Biometry, Heidelberg University, Heidelberg, Germany; Institute of Medical Biometry (IMBI), Department Medical Biometry, Heidelberg University, Heidelberg, Germany; Institute of Medical Biometry (IMBI), Department Medical Biometry, Heidelberg University, Heidelberg, Germany

**Keywords:** additional benefit assessment methods, IQWiG, ESMO, ASCO, simulation study, time-to-event endpoints

## Abstract

**Background:**

For time-to-event endpoints, three additional benefit assessment methods have been developed aiming at an unbiased knowledge about the magnitude of clinical benefit of newly approved treatments. The American Society of Clinical Oncology (ASCO) defines a continuous score using the hazard ratio point estimate (HR-PE). The European Society for Medical Oncology (ESMO) and the German Institute for Quality and Efficiency in Health Care (IQWiG) developed methods with an ordinal outcome using lower and upper limits of the 95% HR confidence interval (HR-CI), respectively. We describe all three frameworks for additional benefit assessment aiming at a fair comparison across different stakeholders. Furthermore, we determine which ASCO score is consistent with which ESMO/IQWiG category.

**Methods:**

In a comprehensive simulation study with different failure time distributions and treatment effects, we compare all methods using Spearman’s correlation and descriptive measures. For determination of ASCO values consistent with categories of ESMO/IQWiG, maximizing weighted Cohen’s Kappa approach was used.

**Results:**

Our research depicts a high positive relationship between ASCO/IQWiG and a low positive relationship between ASCO/ESMO. An ASCO score smaller than 17, 17 to 20, 20 to 24, and greater than 24 corresponds to ESMO categories. Using ASCO values of 21 and 38 as cutoffs represents IQWiG categories.

**Limitations:**

We investigated the statistical aspects of the methods and hence implemented slightly reduced versions of all methods.

**Conclusions:**

IQWiG and ASCO are more conservative than ESMO, which often awards the maximal category independent of the true effect and is at risk of overcompensating with various failure time distributions. ASCO has similar characteristics as IQWiG. Delayed treatment effects and underpowered/overpowered studies influence all methods in some degree. Nevertheless, ESMO is the most liberal one.

**Highlights:**

- For the additional benefit assessment, the American Society of Clinical Oncology (ASCO) uses the hazard ratio point estimate (HR-PE) for their continuous score. In contrast, the European Society for Medical Oncology (ESMO) and the German Institute for Quality and Efficiency in Health Care (IQWiG) use the lower and upper 95% HR confidence interval (HR-CI) to specific thresholds, respectively. ESMO generously assigns maximal scores, while IQWiG is more conservative.
- This research provides the first comparison between IQWiG and ASCO and describes all three frameworks for additional benefit assessment aiming for a fair comparison across different stakeholders. Furthermore, thresholds for ASCO consistent with ESMO and IQWiG categories are determined, enabling a comparison of the methods in practice in a fair manner.
- IQWiG and ASCO are the more conservative methods, while ESMO awards high percentages of maximal categories, especially with various failure time distributions. ASCO has similar characteristics as IQWiG. Delayed treatment effects and under/-overpowered studies influence all methods. Nevertheless, ESMO is the most liberal one. An ASCO score smaller than 17, 17 to 20, 20 to 24, and greater than 24 correspond to the categories of ESMO. Using ASCO values of 21 and 38 as cutoffs represents categories of IQWiG.

## Introduction

A significant phase III clinical trial is not the only step for a successful market authorization application of a new drug but is the most important one, as the quality, safety, and efficacy of the new drug are verified. In case of time-to-event endpoints, a log-rank test is commonly used to investigate if the effect of the new drug against a control treatment is statistically significant. After a significant trial and receipt of approval by regulatory bodies, the new drug’s additional benefit is compared with that of other treatments on the market. With the help of this assessment, the amount of reimbursement for the new drug may be decided and, additionally, patients’ concerns regarding the medical effectiveness and toxicity is reduced.^1^ For time-to-event efficacy endpoints, the question as to whether the new and effective drug provides an additional benefit remains unanswered and is not unequivocally defined. To close this gap, three authorities/societies have developed benefit assessment methods for time to-event endpoints, which can be applied after a significant phase III trial to evaluate the additional benefit.

Firstly, the American Society of Clinical Oncology (ASCO) aims for an assessment of treatment options allowing medical practitioners and patients a shared decision regarding different drugs and their pricing. Therefore, ASCO defines a continuous net health benefit (NHB) score consisting of a clinical benefit score, toxicity score, and bonus points, in which the hazard ratio of the overall survival (OS) point estimate (HR-PE) is used for the clinical benefit score.^2,3^ Secondly, the German Institute for Quality and Efficiency in Health Care (IQWiG) developed a method with ordinal outcome (IQWiG_RR_) using the upper limit of the 95% HR confidence interval (HR-CI) and bonus point adjustments reflecting additionally on toxicity, quality of life, and other important endpoints.^4^ Based on the determined category, the Federal Joint Committee (GBA, germ. Gemeinsamer Bundesaussusch) decides on the additional benefit of the new treatment, which influences the negotiation of the amount of reimbursement between Central Federal Association of Health Insurance Funds (GKV-SV, germ. Spitzenverband Bund der Krankenkassen) and the pharmaceutical company. Thirdly, the European Society for Medical Oncology (ESMO) defined a dual rule considering relative and absolute benefit with ordinal outcome using the lower limit of the 95% HR-CI and the observed absolute difference in median survival times between intervention and control arm (gain).^5,6^ ESMO aims to apply this scoring system to new cancer treatments and spotlights every treatment with the highest score in the ESMO guideline to accelerate the usage.

All three described methods use estimates determined from the clinical trials of the new drug, leading to the question as to which approach provides the best insight for clinical benefit assessment between different drugs in different scenarios. A comparison between ASCO and IQWiG_RR_, hence upper HR-CI and HR-PE estimates, has never been performed. According to ESMO and IQWiG, the upper and lower HR-CI provide more information compared with the HR-PE, due to the variability of the HR-PE. Nevertheless, Büsch et al.^7^ showed that HR-PE might be superior compared with the upper limit of HR-CI estimate and hence a valid alternative. Since ASCO is the only method that grades drugs with a continuous score, it is important to know which ASCO score represents which IQWiG_RR_/ESMO category to compare methods. For ESMO and ASCO, this is partly answered by Cherny et al.^8^ using real studies showing that an ASCO score of 46 or greater defines drugs with substantial benefit (category 4–5) and 41 or less with low benefit (category 1–3). Nevertheless, this was done using only 102 studies applying only ESMO and ASCO, and hence, neither focused on the statistical part of the methods nor included IQWiG_RR_ in the comparison. Thus, we provide the first comparison of all three additional benefit assessment methods (ABAM) within one comprehensive simulation study.

Even though the purpose of the three methods is partly different, their main intent is the unbiased comparison of effective treatments. Thus, it is important to know how the different methods assess various treatment effects and how they are associated for a fair comparison. Since the main difference between the methods is the use of different clinical benefit estimates (HR-PE, HR-CI), we focus on the comparison of the statistical aspects of the methods. Furthermore, we answer the question as to which ASCO score corresponds to which ESMO/IQWiG category. Lastly, we verify the association between ASCO and ESMO provided by Cherny et al.^8^ and compare it to others in the research literature, which calculated correlations between those two methods.^9–11^

## Methods

The benefit assessment methods and the approach of the simulation study are presented below. As two versions of IQWiG method are considered, four methods are compared in total denoted by ASCO, IQWiG_RR_, Mod-IQWiG_HR_, and ESMO. The methods are applied after a statistically significant phase III trial based on the log-rank test. We restrict the application to single phase III trials with OS as the primary endpoint and do not consider cases in which two or more phase III trials are needed for market authorization. To achieve a fair comparison for the statistical aspects of the method in an OS/advanced diseases framework, only the clinical benefit and tail of the curve bonus points of ASCO was assessed. Similar considerations apply to ESMO so that categories 1 to 4 are considered in our simulation study. Hence, we implemented a slightly reduced version of all methods to focus on the statistical aspect of each method. In addition, we focus on OS as the main primary endpoint in oncology trials. For other time-to-event endpoints (e.g., progression-free-survival), the benefit assessment is slightly different, as the methods penalize these endpoints as they are not as reliable and precise as OS. However, the main aspect of statistical quantity used by the method does not change, so the results are generalizable.

### Additional Benefit Assessment Methods (ABAM)

**Figure 1:**
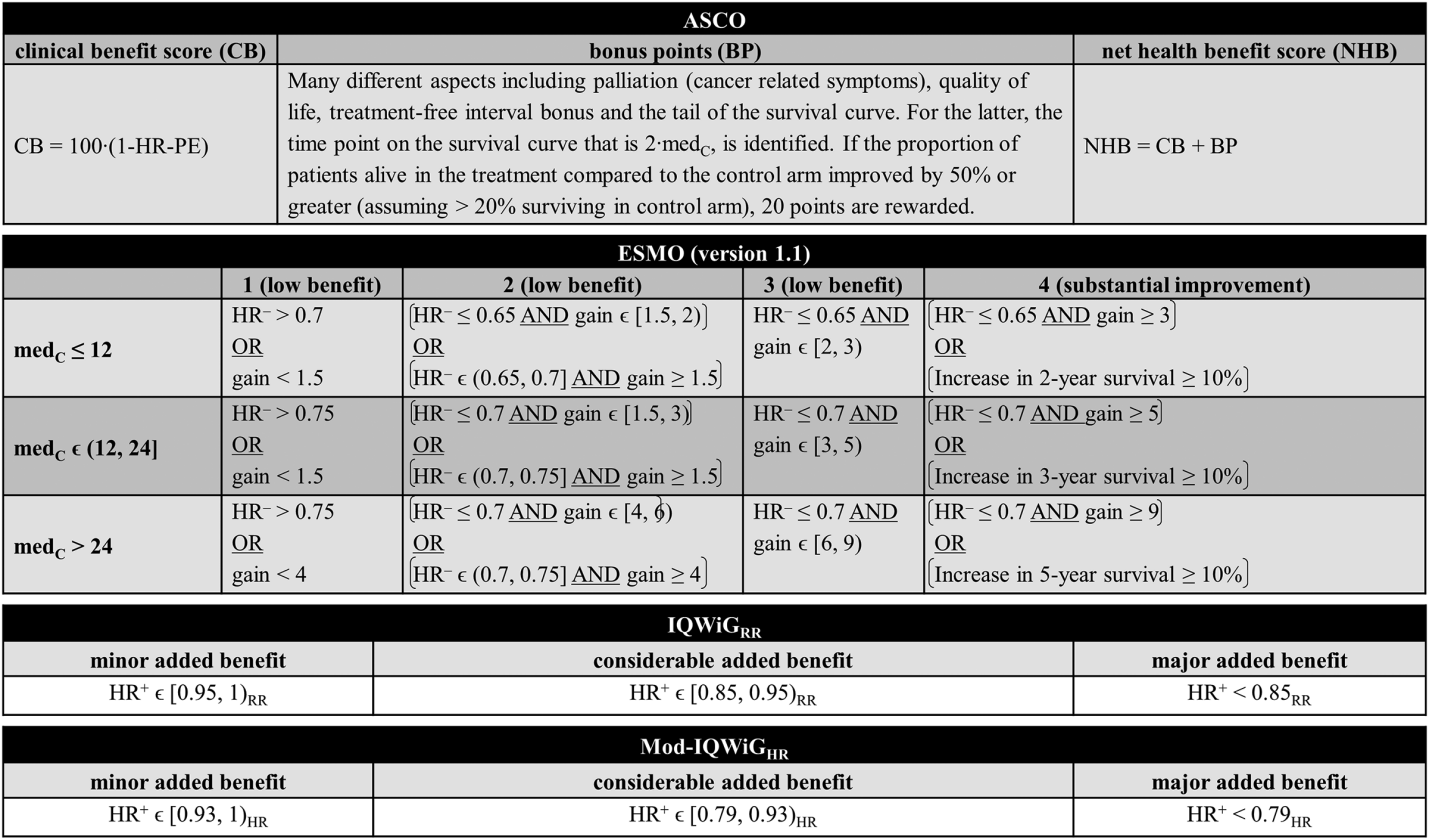
Detailed overview of ASCO, ESMO, IQWiG_RR_, and Mod-IQWiG_HR_ for overall survival/advanced diseases framework focusing on the statistical aspects of each method (modified from Büsch et al.^7^). ASCO, American Society of Clinical Oncology; BP, bonus points; CB, clinical benefit score; ESMO, European Society for Medical Oncology; gain, estimated absolute difference in median survival times (in months); HR, hazard ratio; HR^+^, estimated upper 95% confidence interval limit of the hazard ratio; HR^–^, estimated lower 95% confidence interval limit of the hazard ratio; HR-PE, hazard ratio point estimate; IQWiG, Institute for Quality and Efficiency in Health Care; IQWiG_RR_, original IQWiG method; med_C_, median survival time in the control group (in months); Mod-IQWiG_HR_, modified IQWiG method using upper confidence interval limit based on IQWiG RR-scaled thresholds (transformation into HR-scaled thresholds using the conversion formula proposed by VanderWeele^12^); NHB, net health benefit score; RR, relative risk.

Figure 1 provides a detailed overview of the construction of the statistical aspects of each method used in the simulation study.

1. ASCO uses a sum of a clinical benefit score and bonus points to calculate the NHB score. As the main component, the NHB defines the clinical benefit score, which uses the HR-PE to calculate a continuous value: 100ċ(1 − HR-PE). The bonus points part consists of many different aspects including the tail of the survival curve. Here, the time point on the survival curve that is two times the median OS of the control arm (2ċmed_C_) is identified. If the proportion of patients alive in the treatment compared with the control arm improved by 50% or more (assuming >20% surviving in control arm), 20 points are rewarded.
2. IQWiG_RR_ evaluates the additional benefit of new drugs using the upper limit of the HR-CI (HR^+^) and bonus point adjustments grading drugs into three categories (major, considerable, and minor added benefit). For the main classification, the HR^+^ estimates are compared with relative risk (RR)–scaled thresholds 0.85 and 0.95. Thus, HR^+^ <0.85 is considered as major, 0.85 ≤ HR^+^ < 0.95 as considerable, and HR^+^≥ 0.95 as a minor added benefit.
3. In addition, as proposed by Büsch et al.,^7^ we transform the RR-scaled IQWiG_RR_ thresholds with VanderWeele conversion formula^12^ into HR-scaled thresholds (Mod-IQWiG_HR_), i.e., 0.79 and 0.93.
4. ESMO has developed a combination of relative benefit using the lower limit of the 95% HR-CI (HR^–^), absolute benefit using the gain definition, and bonus point adjustments. These estimates are compared with specific thresholds leading to an ordinal rating for the classification with 4 categories, where grade 4 represents substantial and grades 3 to 1 low benefit. Grade 4 can already be achieved if the survival rate increases by ≥10% at key milestones.

### Simulation Study

**Figure 2:**
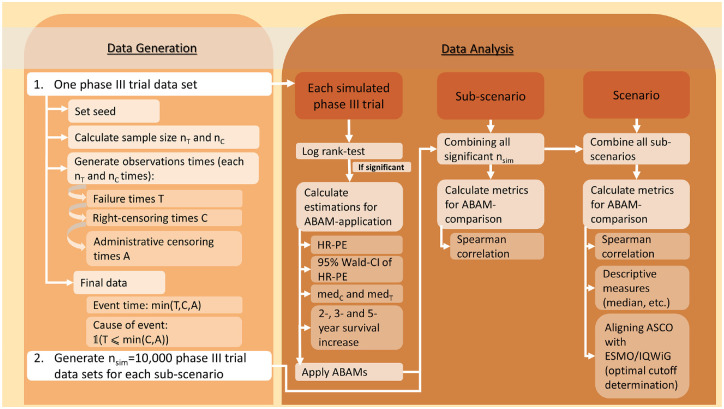
Flowchart of the simulation study process. ABAM, additional benefit assessment method; ASCO, American Society of Clinical Oncology; CI, confidence interval; HR, hazard ratio; HR-PE, hazard ratio point estimate; IQWiG, Institute for Quality and Efficiency in Health Care; med_C_, median survival time in the control group (in months); med_T_, median survival time in the treatment group (in months); n_C_, sample size of control group; n_sim_, number of simulation runs; _T_, sample size of treatment group.

To answer the research question, a comprehensive simulation study was performed with its process visualized in Figure 2.

#### Simulation Setup

We simulated phase III clinical trials comparing one treatment against one control arm with a 1:1 allocation ratio. In addition, a combination of administrative censoring—censoring a patient with an event after end of study—with an accrual time of two years and follow-up time of 2 · med_C_ as well as independent exponential censoring was used, aiming for an overall censoring rate of 60%. We distinguish between the true treatment effect (trueHR), which is used for the data generation, and the design treatment effect (designHR), which is assumed for sample size calculation, and introduce HR_var_, which measures the deviance between designHR and trueHR such that trueHR = designHR · HR_var_. Hence, this definition causes scenarios with incorrect assumed treatment effects leading to overpowered (HR_var_ > 1) and underpowered (HR_var_ < 1) studies. As a full picture of potential aspects should be examined, a large range of treatment effects was chosen (designHR ϵ {0.3, 0.32, . . ., 0.9}). Thus, this includes large treatment effects, which are rarely reported but nevertheless exist.^13–15^ Furthermore, to ensure realistic simulated phase III trials, sample size calculation using Schoenfeld’s approach^16,17^ was performed to achieve a specific power for a two-sided log-rank test at a significance level of 5% assuming treatment effect designHR.

**Figure 3:**
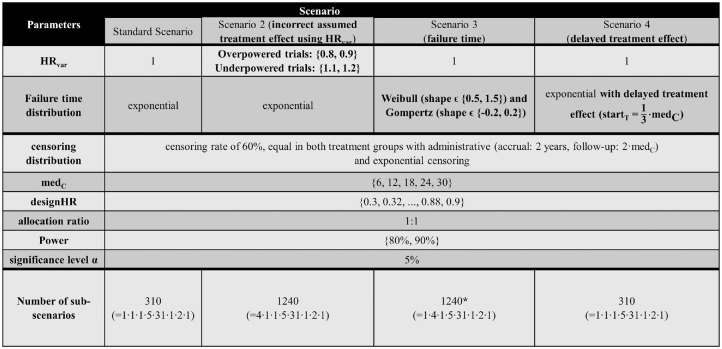
Overview of all simulation scenarios including the parameters, their distinctions, and the resulting number of subscenarios. Differences from the standard scenario regarding the parameter choice are highlighted in bold. *Some subscenarios of scenario 3b (Gompertz) resulted in an administrative censoring rate larger than the targeted 60%. To maintain comparable scenarios, these subscenarios were excluded, leading to 1,184 subscenarios instead of 1,240. designHR, design hazard ratio, used for sample size calculation; HR, hazard ratio; med_C_, median survival time in the control group (in months); HR_var_, factor for deviance between designHR and trueHR; start_T_, time point at which the treatment effect sets in; trueHR, true underlying hazard ratio for data generation.

The failure time generation was carried out for exponential, Weibull and Gompertz distributions with proportional hazards. Thus, the shape parameter was fixed for Weibull and Gompertz, causing the hazard function to increase/decrease over time. The corresponding parameters of the failure time distributions were obtained by fixing med_C_ and trueHR. The implemented scenarios are the same as in Büsch et al.^7^ Each scenario consists of multiple parameter combinations (subscenarios) each with n_sim_ = 10,000 simulation runs. In the following, an overview of all scenarios with the respective subscenarios is given (Figure 3):

- *Standard scenario:* exponentially distributed failure times using HR_var_ = 1, med_C_ϵ {6, 12, 18, 24, 30 months}, designHR ϵ {0.3, 0.32, . . ., 0.9}, power ϵ {80%, 90%}, leading to (5ċ31ċ2=) 310 subscenarios.
- *Incorrect assumed treatment effect (scenario 2):* Overpowered/underpowered studies using the same parameters as the standard scenario, except HR_var_ϵ {0.8, 0.9, 1.1, 1.2}, leading to (4ċ5ċ31ċ2 =) 1,240 subscenarios.
- *Different parameter distributions (scenario 3):* Standard scenario with Weibull and Gompertz instead of exponential failure time distributions, while adhering to proportional hazards.
  - *Scenario 3a:* Weibull using shape ϵ {0.5, 1.5}, leading to (5ċ31ċ2ċ2 =) 620 subscenarios
  - *Scenario 3b:* Gompertz using shape ϵ {−0.2, 0.2}, leading to (5ċ31ċ2ċ2 =) 620 subscenarios. Some subscenarios with decreasing hazards resulted in an administrative censoring rate larger than the targeted 60%. To still have comparable scenarios, these subscenarios were excluded (see Figure 3 and Appendix for further information).
- *Delayed treatment effect (scenario 4):* Delayed treatment effect for the treatment group, which is a type of nonproportional hazards, using piecewise exponential failure time distributions, leading to (31ċ5ċ2 =) 310 subscenarios. To achieve a late treatment effect for the treatment group, a piecewise exponential distribution was chosen:

$$\begin{array}{l} F_{C}\left(x\right)=1-\exp(-\lambda_{C}\cdot x)\\ F_{T}\left(x\right)=\{\begin{array}{cc} \mathrm{1-exp}\left(-\lambda_{C}\cdot x\right), & x\in [0,\;{\mathrm{start}}_{T}]\\ 1-\exp(-\lambda_{C}\cdot {\mathrm{start}}_{T}\cdot \exp\left(-\lambda_{T}\cdot \left({x-\mathrm{start}}_{T}\right)\right), & \mathrm{otherwise} \end{array} \end{array}$$

where F_C_ and F_T_ are the cumulative distribution functions of the treatment and control group, λ_C_ > 0 and λ_T_ > 0 are the parameters of the corresponding exponential distributions, and start_T_ $=\frac{1}{3}\cdot \mathrm{me}d_{C})$ is the time point at which the treatment effect sets in. The failure times of the treatment group were generated using the inversion method by Kolonko (chapter 8).^18^ Hence, proportional hazards were assumed before and after start_T_. In addition, λ_C_ and λ_T_ were defined the same way as in the standard scenario (see the Appendix for further information).

For a method comparison under realistic circumstances, the subscenarios were combined, meaning the complete range of designHRs, HR_var_, power, med_C_, and shape of each scenario were used together. Moreover, the standard scenario and scenario 3a and 3b were also combined for a situation in which all different failure time distributions are present. The Appendix gives more details about the ADEMP (aims, data-generation mechanism, estimands, methods and performance) measures structure proposed by Morris et al.^19^ used in our simulation study.

#### Data Analysis

For the subsequent application of the methods, the HR-PE with corresponding 95% Wald-CI and the 2-, 3-, and 5-y survival increase were required. In addidion, for ASCO bonus point adjustment, the “tail of the curve” and ESMO absolute benefit rule median OS of the control (med_C_) or treatment arm (med_T_) had to be calculated. However, if the survival curve does not fall below 50% (e.g., due to large treatment effects), the median survival time cannot be observed. As in Büsch et al.,^7^ a conservative approach was implemented, using the last observed censoring or event time point of the survival curve instead.

As the main metric for the assessment of the relationship between ASCO and ESMO/IQWiG_RR_/Mod-IQWiG_HR_, the pairwise Spearman correlation with the interpretation provided by Mukaka was used.^20,21^ In addition, descriptive measures including median and absolute and relative frequencies were used to describe the methods differences. For graphical illustrations, box plots, heat maps, and line charts were generated.

To investigate which ESMO/IQWiG_RR_/Mod-IQWiG_HR_ category corresponds to which ASCO score, maximizing the weighted Cohen’s Kappa κ approach was used for cutoff value determination^22,23^:

$$\kappa =1-\frac{\sum_{i=1}^{k}\sum_{j=1}^{k}w_{\mathrm{ij}}\cdot x_{\mathrm{ij}}}{\sum_{i=1}^{k}\sum_{j=1}^{k}w_{\mathrm{ij}}\cdot m_{\mathrm{ij}}},$$

where *i* = 1, . . ., *k* and *j* = 1, . . ., *k* are the methods categories, *x* is the observed probability matrix, *w* is the quadratic weights matrix, and *m* is the expected probability matrix. Because ESMO, IQWiG_RR_, and Mod-IQWiG_HR_ are ordinal scores, disagreements close to the diagonal imply a smaller disagreement than those far from the diagonal. Thus, quadratic Fleiss-Cohen weights were used.

### Sensitivity Analysis

As a sensitivity analysis for relationship assessment, Kendall-τ_b_ was calculated. Furthermore, for optimal cutoff determination, the receiver-operating characteristic (ROC) curves were used dividing categories pairwise and considered optimal when the point on the ROC curve is closest to the point (0,1). As second sensitivity analysis Svenssons method^24,25^ was used, which defines cutoffs where marginal distribution of ordinal method and continuous ASCO are the same.

#### Software

The simulation study was performed using the software R^26^ version 4.2.1, with packages “ggplot,”^27^“survival,”^28,29^“flexsurv,”^30^“cutpointr,”^31^“vcd,”^32^ and “pcaPP.”^33^ R-Code and results of the sensitivity analyses are available at github.com/cbuesch/ASCOvsIQWiGvsESMO.

## Results

**Figure 4:**
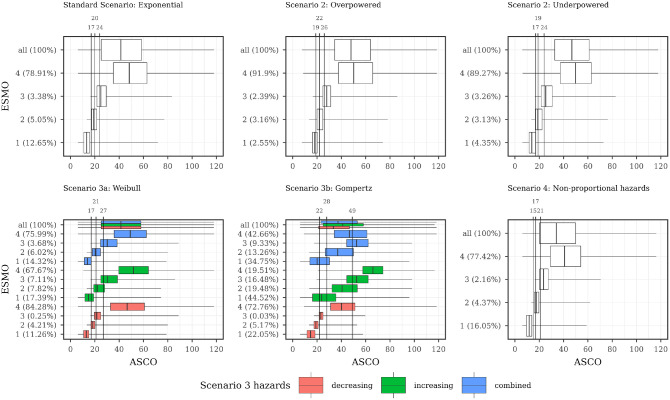
Description of ASCO and ESMO illustrated using boxplots for all scenarios with combined subscenarios, meaning the complete range of designHRs, HR_var_, power, med_C_, and shape was used together. In addition, vertical lines are added, showing the ASCO cutoff values consistent with ESMO categories using the maximizing weighted Cohen’s Kappa approach. ASCO, American Society of Clinical Oncology; designHR, design hazard ratio, used for sample size calculation; ESMO, European Society for Medical Oncology; HR, hazard ratio; HR_var_, factor for deviance between designHR and trueHR; med_C_, median survival time in the control group (in months); trueHR, true underlying hazard ratio for data generation.

**Figure 5:**
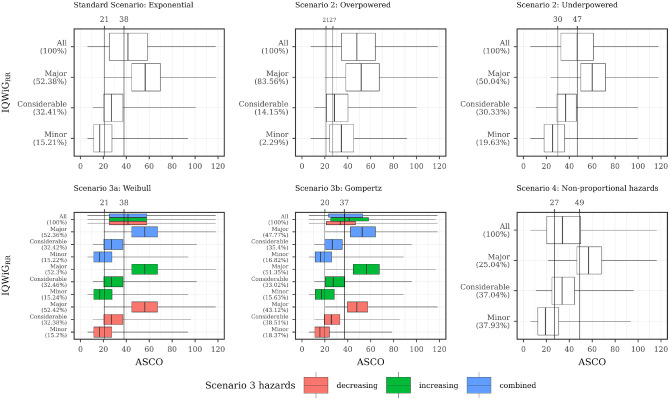
Description of ASCO and IQWiG_RR_ illustrated using boxplots for all scenarios with combined subscenarios, meaning the complete range of designHRs, HR_var_, power, med_C_, and shape was used together. In addition, vertical lines are added, showing the ASCO cutoff values consistent with IQWiG_RR_ categories using the maximizing weighted Cohen’s Kappa approach. ASCO, American Society of Clinical Oncology; designHR, design hazard ratio, used for sample size calculation; HR, hazard ratio; HR_var_, factor for deviance between designHR and trueHR; IQWiG, Institute for Quality and Efficiency in Health Care; IQWiG_RR_, original IQWiG method; medC, median survival time in the control group (in months); trueHR, true underlying hazard ratio for data generation.

**Figure 6:**
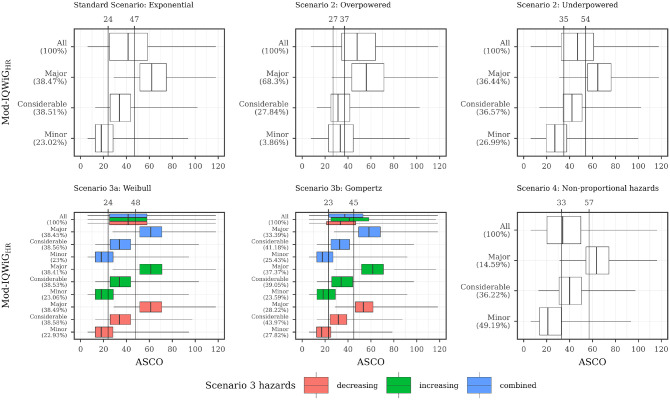
Description of ASCO and Mod-IQWiG_HR_ illustrated using boxplots for all scenarios with combined subscenarios, meaning the complete range of designHRs, HR_var_, power, med_C_, and shape was used together. In addition, vertical lines are added, showing the ASCO cutoff values consistent with Mod- IQWiG_HR_ categories using the maximizing weighted Cohen’s Kappa approach. ASCO, American Society of Clinical Oncology; designHR, design hazard ratio, used for sample size calculation; HR, hazard ratio; HR_var_, factor for deviance between designHR and trueHR; IQWiG, Institute for Quality and Efficiency in Health Care; Mod-IQWiG_HR_, modified IQWiG method using upper confidence interval limit based on IQWiG RR-scaled thresholds (transformation into HR-scaled thresholds using the conversion formula proposed by VanderWeele^12^); med_C_, median survival time in the control group (in months); trueHR, true underlying hazard ratio for data generation.

**Figure 7:**
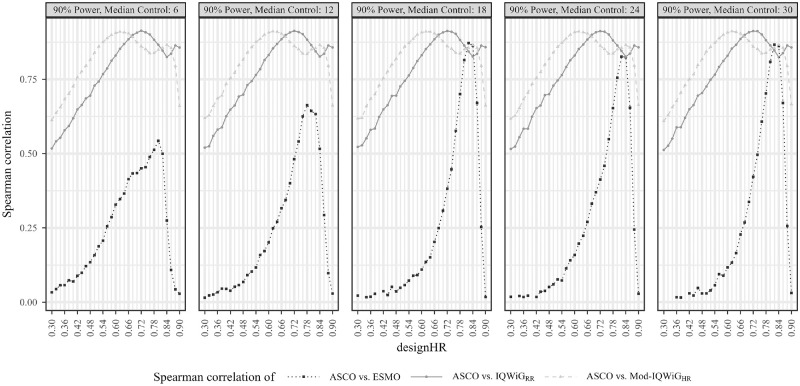
Pairwise Spearman correlation results between ASCO and ESMO/IQWiG_RR_/Mod-IQWiG_HR_ illustrated using line charts for the standard scenario (designHR = trueHR) with different underlying median survival times for the control group (6, 12, 18, 24, and 30 months), designHRs (0.3 to 0.9), censoring rate of 60%, and power of 90%. In subscenarios with very large treatment effects, the ordinal additional benefit assessment methods (IQWiG_RR_, Mod-IQWiG_HR_, and ESMO) only assigned the same category; therefore, some correlations could not always be computed and hence are missing (e.g., designHR < 0.42, right panel). ASCO, American Society of Clinical Oncology; designHR, design hazard ratio, used for sample size calculation; ESMO, European Society for Medical Oncology; HR, hazard ratio; IQWiG, Institute for Quality and Efficiency in Health Care; IQWiG_RR_, original IQWiG method; med_C_, median survival time in the control group (in months); Mod-IQWiG_HR_, modified IQWiG method using upper confidence interval limit based on IQWiG RR-scaled thresholds (transformation into HR-scaled thresholds using the conversion formula proposed by VanderWeele^12^); trueHR, true underlying hazard ratio for data generation.

**Figure 8:**
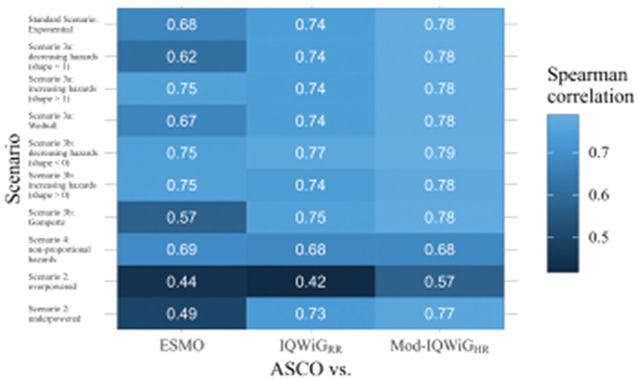
Pairwise Spearman correlation results between ASCO and ESMO/IQWiG_RR_/Mod-IQWiG_HR_ method illustrated using a heat map for all scenarios with combined subscenarios, meaning the complete range of designHRs, HR_var_, power, med_C_, and shape was used together. ASCO, American Society of Clinical Oncology; designHR, design hazard ratio, used for sample size calculation; ESMO, European Society for Medical Oncology; HR, hazard ratio; HR_var_, factor for deviance between designHR and trueHR; IQWiG, Institute for Quality and Efficiency in Health Care; IQWiG_RR_, original IQWiG method; med_C_, median survival time in the control group (in months); Mod-IQWiG_HR_, modified IQWiG method using upper confidence interval limit based on IQWiG RR-scaled thresholds (transformation into HR-scaled thresholds using the conversion formula proposed by VanderWeele^12^); trueHR, true underlying hazard ratio for data generation.

The relation between ASCO/ESMO, ASCO/IQWiG_RR_, and ASCO/Mod-IQWiG_HR_ for different scenarios is displayed using boxplots in Figures 4 to 6, respectively. A further description of the relation between methods for different subscenarios of the standard scenario is displayed using pairwise Spearman correlation in Figure 7. In addition, Figure 8 shows pairwise Spearman correlation between ASCO and the other methods for all scenarios.

### Association of ESMO, ASCO, IQWiG_RR_, and Mod-IQWiG_HR_ Scoring

#### Standard scenario

ESMO mainly assigns the maximal score (78.91%, Figure 4 top left panel) while IQWiG_RR_ (52.38%, Figure 5 top left panel) and Mod-IQWiG_HR_ (38.47%, Figure 6 top left panel) have reduced rates. In addition, ASCO has a median score of 41. Furthermore, an ASCO median score of 13, 19, 25, and 48 is present for ESMO categories and 17, 27, and 56 for IQWiG_RR_ categories, leading to a visually better separation for IQWiG_RR_ than for ESMO. As ESMO has more categories than IQWiG_RR_ and Mod-IQWiG_HR_, natural differences between ESMO categories are less pronounced. The comparison between ASCO/IQWiG_RR_ and ASCO/Mod-IQWiG_HR_ using pairwise Spearman correlation (Figure 7) shows a moderate to very high positive correlation, with a maximal correlation of 0.91 over the range of treatment effects. ASCO/ESMO reveals negligible to low correlations for nearly all designHRs except for “moderate” treatment effects with a designHR of about 0.80 where a similar correlation as between ASCO and IQWiG_RR_ is achieved (Figure 7). These findings are supported when combing all subscenarios, resulting in a Spearman correlation of 0.68, 0.74, and 0.75 for ASCO/ESMO, ASCO/IQWiG_RR_, and ASCO/Mod-IQWiG_HR_ comparison (Figure 8). Furthermore, the achieved correlation peak between ASCO and ESMO at moderate treatment effects is substantially lower with low med_C_ (Figure 7, left two panels); for example, for med_C_ = 6, the maximal correlation is only 0.54 instead of 0.87 for med_C_ = 30.

The only difference between ASCO/IQWiG_RR_ and ASCO/Mod-IQWiG_HR_ correlation comparisons is the shifted correlation maximum to greater treatment effects due to smaller threshold values for Mod-IQWiG_HR_ compared with IQWiG_RR_ (Figure 7); that is, for a maximal score, HR^+^ must be smaller than 0.85 (IQWiG_RR_) or 0.79 (Mod-IQWiG_HR_), respectively. The correlation between ASCO/Mod-IQWiG_HR_ reaches its maximum at a designHR of 0.62 (for all med_C_ values), instead of 0.72 for IQWiG_RR_. This can be explained by the IQWiG_RR_/Mod-IQWiG_HR_ classification solely depending on the HR^+^ estimate, which leads to the most uniform category distribution at a designHR of 0.72 or 0.62, respectively.

#### Scenario 2 (incorrect assumed treatment effect)

Overpowered studies have increased scores or maximal category rates for all methods (Figures 4–6). In addition, all correlations between ASCO and ESMO/IQWiG_RR_/Mod-IQWiG_HR_ drop to a low positive value (Figure 8). Underpowered studies lead to similar category proportions for IQWiG_RR_ and Mod-IQWiG_HR_, while ESMO and ASCO are influenced by underpowered studies in a similar way as in overpowered studies (Figures 4–6). Similar findings are present for the Spearman correlation, where the IQWiG_RR_/Mod-IQWiG_HR_ comparison to ASCO is still highly positive for underpowered studies and hence remains similar to the standard scenario, while the ESMO and ASCO correlation drops down to a low positive value as in overpowered studies.

ASCO awards higher values for overpowered (median score = 48) and underpowered (=47) scenarios compared with the standard scenario. ESMO’s maximal category proportion increases from 78.91% in the standard scenario to about 90% for over- and underpowered studies, which can also be seen in the low correlation value of 0.44 and 0.49 for over- and underpowered studies.

IQWiG_RR_ is only affected by overpowered studies, leading to a higher proportion of maximal scores (83.56%) and smaller proportions in the other 2 categories (Figure 5). Using HR-scaled instead of RR-scaled thresholds of Mod-IQWiG_HR_ reduces this increase in the maximal score (68.30%, Figure 6). This behavior can also be seen for the correlation, where ASCO and Mod-IQWiG_HR_ still show a moderate correlation of 0.57, while for ASCO and IQWiG_RR_, only a low correlation of 0.42 is present. A reason for the low correlation for ASCO/IQWiG_RR_ is the similar ASCO score for IQWiG_RR_ categories “considerable” and “minor” (Figure 5; middle top panel).

In case of underpowered studies, IQWiG_RR_ and Mod-IQWiG_HR_ have similar category distributions as in the standard scenario, leading to similar correlation values as well.

#### Scenario 3 (Different parameter distributions)

Underlying failure time distributions hardly influence the outcome of IQWiG_RR_, Mod-IQWiG_HR_, and ASCO, illustrated by almost no change in category proportions for IQWiG_RR_ and Mod-IQWiG_HR_ (Figures 5 and 6), similar ASCO median scores (scenario 3a: 41; scenario 3b: 37) and the same Spearman correlations between ASCO/IQWiG_RR_ and ASCO/Mod-IQWiG_HR_ (Figure 8).

However, Gompertz failure time distribution affects ESMO (scenario 3b), for example, category 1 proportion with exponential, Weibull and Gompertz distribution is 12.65%, 14.32% and 34.75%, respectively (Figure 4). The correlation between ASCO and ESMO is influenced only in case of Gompertz distributed failure times (Figure 8).

#### Scenario 4 (delayed treatment effect)

Delayed treatment effects lead to a reduced score for ASCO and a shift of category proportions for the ordinal methods compared with proportional hazards. The exception is the ESMO method, in which a delayed treatment effect does not show any influence. For example, ASCO has a reduced median score of 34 (v. 41) and IQWiG_RR_ has a reduced maximal score of 25.04% (v. 52.38%) and an increased minimal score of 37.93% (v. 15.21%) compared with the standard scenario (Figure 5). Mod-IQWiG_HR_ has a similar shift, that is, 38.47% to 14.59% and 23.02% to 49.19% for the maximal and minimal score compared with the standard scenario (Figure 6). ESMO, however, has similar category proportions of 77.42% (v. 78.91%), 2.16% (v. 3.38%), 4.37% (v. 5.05%), and 16.05% (v. 12.65%) for categories 1, 2, 3, and 4 compared with the standard scenario (Figure 4). This is substantiated by the correlation results, showing a reduction for ASCO/IQWiG_RR_ and ASCO/Mod-IQWiG_HR_ compared with the standard scenario, while the correlation of ASCO/ESMO is not affected (Figure 8).

Similar results can be seen using Kendall-τ_b_ instead of Spearman correlation (see the Appendix and github repository). The only difference is that the Kendall-τ_b_ results show overall smaller values than the Spearman correlation does.

##### Determining which ASCO cutoff values are consistent with ESMO, IQWiG_RR_, and Mod-IQWiG_HR_ categories

Figures 4 to 6 also depict ASCO cutoff values for different scenarios, which are consistent with ESMO, IQWiG_RR_, and Mod-IQWiG_HR_ categories using the maximizing weighted Cohen’s Kappa approach. In case of proportional hazards (Figures 5 and 6; standard scenario, scenario 3) for IQWiG_RR_ and Mod-IQWiG_HR_, similar ASCO cutoff values can be observed. The cutoff values indicate that ASCO < 21 equals the IQWiG_RR_ category “minor,” 21 ≤ ASCO < 38 the category “considerable,” and ASCO ≥ 38 is equivalent to the category “major.” Scenario 3a has the same cutoff values, while in scenario 3b, the cutoff values are 20 and 37 (Figure 5). ESMO shows increased cutoff values for ASCO of 22, 28, and 49 in case of scenario 3b (Gompertz failure times) compared with 17, 20, and 24 in the standard scenario (exponential failure times).

If a delayed treatment effect is present or a study is underpowered, cutoff values increase for IQWiG_RR_ and Mod-IQWiG_HR_ compared with the standard scenario. Overpowered studies, however, show a decreased cutoff value for defining the maximal category “major,” while the other cutoff value stays very similar compared with the standard scenario (Figures 5 and 6; middle upper panel). Cutoff values consistent with ESMO remain similar over the different scenarios (Figure 4). This is caused by a high proportion of maximal scoring in ESMO, which leads to correct classification in most cases when the upper cutoff value is chosen low enough, and thus, the incorrectly classified categories 2 and 3 do not play a major role in the overall classification.

Overall, ASCO provides visually better separation between the categories of IQWiG_RR_ (Figure 5) and Mod-IQWiG_HR_ (Figure 6), while ESMO categories 1, 2, and 3 have quite similar ASCO scores (Figure 4). This also reflects on the ASCO cutoff values. Cutoff values for ESMO categories are quite similar, that is, 17, 20, and 24 in the standard scenario (exponential failure times), while for IQWiG_RR_ and Mod-IQWiG_HR_, the cutoff values are further apart, that is, 21 and 38 as well as 24 and 47, respectively. Furthermore, ASCO cutoff values representing IQWiG_RR_ and Mod-IQWiG_HR_ change if assumptions such as proportional hazards (e.g., delayed treatment effect) are violated or if wrong sample sizes, due to wrongly assumed treatment effects, are present. With different underlying failure time distributions, however, the cutoff values stay similar. Cutoff values representing ESMO categories remain similar in most of the scenarios.

## Discussion

In our research, we performed an extensive simulation study including different failure time distributions and treatment effects and further investigated which statistical measure is the most appropriate for additional benefit assessments. Thus, the knowledge gap of differences between ASCO, IQWiG_RR_, and ESMO is further diminished.

We clearly show that ASCO/Mod-IQWiG_HR_ always provides a stronger positive relationship than or equal to the ASCO/IQWiG_RR_ relationship. Nevertheless, a high positive relationship between ASCO/IQWiG_RR_ and ASCO/Mod-IQWiG_HR_ is present, while the ASCO/ESMO relationship provides only a low positive relationship. Only moderate treatment effects lead to similar results in subscenarios of med_C_ ≥ 18 and hence higher correlation between ASCO/ESMO, which is similar to ESMO/IQWiG_RR_ illustrated by Büsch et al.^7^ The result of a moderate correlation between ASCO/ESMO is very similar to a real study application performed by Cherny et al.,^8^ which showed a correlation of 0.68. Other previous comparisons based on real studies showed correlations of 0.17,^9^ 0.397,^10^ or 0.40,^11^ which cannot be verified in our research. Besides that, the mentioned literature shows inconsistent correlation values. Another reason for this is the application on real studies and the implementation of the full methods. Since we tried to achieve a fair comparison for the statistical aspects of the four ABAMs, we focused on the quantitative characteristics.

If trials are over-/underpowered due to over-/underestimated treatment effects in the sample size calculation, ESMO maximal category percentage increases. ASCO has an increased and decreased score for overpowered and underpowered studies, respectively. IQWiG_RR_ and Mod-IQWiG_HR_, however, show a conservative behavior for underpowered studies, meaning similar results as in the standard scenario are present. In case of overpowered studies, IQWiG_RR_ shows a larger increased maximal category than ESMO does, thus showing a more conservative behavior. However, because ESMO already has a high percentage of maximal scores in the standard scenario, it cannot increase much further, whereas IQWiG_RR_ has more room to increase. To achieve a higher additional benefit grading, a study may be deliberately overpowered. To prevent this, method application should be closely monitored. However, in real studies, true power is not known because the true underlying treatment effect is unknown. Hence, the ABAMs cannot adequately penalize wrongly powered studies. Thus, it is even more important to choose the assumed treatment effect carefully for sample size planning. Mod-IQWiG_HR_ provides less shifted proportions in categories and is a more conservative solution in case of overpowered studies, but it still shows an increased maximal scoring percentage.

Moreover, IQWiG_RR_ and Mod-IQWiG_HR_ are hardly influenced by different failure time distributions; hence, similar category proportions are present as in the standard scenario, which is favorable since proportional hazards are still present. ESMO, on the contrary, is influenced by Gompertz failure time distribution, showing a shifted proportion to the minimal category, whereas ASCO is only slightly influenced.

If a delayed treatment effect is present, ESMO has a slightly shifted proportion to the minimal category compared with the standard scenario, while all other methods show a more drastic shift or a reduced score, which is desirable considering the assumption of the Cox regression model is not fulfilled. This illustrates the more liberal behavior of ESMO compared with the other methods in case of delayed treatment effects. Note that, like in Büsch et al.,^7^ only situations with med_T_ >> med_C_ have been considered to avoid punishing ESMO for its design.

Our simulation study further calculated the optimal cutoff values for ASCO, which correspond to ESMO and IQWiG categories. With these results, it is now possible to compare ASCO scores with ESMO/IQWiG_RR_/Mod-IQWiG_HR_ (and vice versa) without the need to apply all methods. Cutoff values that differ between scenarios are a sign of different method behavior due to changed settings. ASCO provides visually better separation between the categories of IQWiG_RR_ and Mod-IQWiG_HR_ leading to cutoff values, which are further apart for IQWiG_RR_ and Mod-IQWiG_HR_ compared with ESMO. Since ESMO comprises more categories than IQWiG_RR_ and Mod-IQWiG_HR_, natural differences between ESMO categories are less pronounced. An ASCO score of ≤17, between 17 and 20, between 20 and 24 and ≥24 correspond to ESMO categories, while ASCO values of 21 and 38 as cutoffs represent IQWiG_RR_ categories. With various underlying failure time distributions such as Weibull and Gompertz still adhering to the proportional hazard assumption, ESMO tends to be more susceptible, making the determination of consistent cutoff values for ASCO representing ESMO categories not possible. Furthermore, the ESMO maximal score can be achieved over almost all ASCO values, resulting in similar cutoff values, illustrating again the liberal behavior of ESMO. These cutoff values for ESMO, however, are not consistent with the results of Cherny et al.,^8^ who used 102 randomized controlled trials instead of a simulation study, resulting in an ASCO score of 46 or greater and 41 or less to define substantial benefit (category 4) and low benefit (category 1–3). A reason for the different cutoff values is the focus on the statistical aspects of the methods in our simulation study, while Cherny et al.^8^ used the full method with bonus point adjustments. Future research should further focus on the verification of our cutoff values in real-world data, using all aspects of the ABAMs for a realistic interpretation of the cutoff values.

The possible bias using HR-PE estimates^4^ and the concern that the use of the lower CI limit could lead to a larger probability of higher grades^34,35^ could not be confirmed. The choice for each threshold has a greater influence on the probability of higher categories. This aspect was also examined by Büsch et al. They pointed out that the currently used thresholds of ESMO are too liberal and lead to a high false-positive rate, resulting in an easily achievable maximal category.^7^

Furthermore, ESMO does not penalize, with a reduced percentage of the maximal category when different assumptions are violated, such as proportional hazards (e.g., delayed treatment effect) or over-/underpowered studies. This finding contradicts the results by Dafni et al.,^36^ who stated that ESMO does not show discriminatory behavior in over-/underpowered trials. However, this can be the result of using different ESMO versions (Dafni: v1.0) and different parameters ranges in the simulation study.

One limitation of our research is that we investigated only the statistical aspect of the methods and hence implemented a slightly reduced version of all methods. In addition, we implemented only one kind of nonproportional hazards (scenario 4: delayed treatment effect). Thus, further research is needed. Moreover, the validity examination of the performed comparison was not assessed because no gold standard method defining a true additional added benefit exists (i.e., a definition of a treatment deservedly classified as maximal category is missing). An investigation defining different underlying trueHR as deserved maximal category by the ABAM was performed by Büsch et al.,^7^ showing that the lower CI has the best ROC and AUC values. Nevertheless, we show that ESMO, which uses the lower CI limit, still has an inappropriate rate of maximal scores. Hence, to improve the assessment of additional benefit, our results can be used as a guide for updates and/or modifications of additional benefit methods. Nevertheless, future research may focus on the definition of a true additional benefit of a treatment. One possibility would be to ask patients what they feel is a real additional benefit. However, this is not the aim of the present article, since we compare the existing ABAMs, which use the specific measurements described in our article.

## Conclusions

ASCO and IQWiG_RR_ as well as ASCO and Mod-IQWiG_HR_ additional benefit methods show a high positive association; hence, similar scoring distributions are present. Our research clearly reinforces that IQWiG_RR_ is more conservative than ESMO in most scenarios. Furthermore, ASCO has similar characteristics as IQWiG_RR_ and also shows more conservative behavior. Delayed treatment effects and under-/overpowered studies influence all methods to some degree. Nevertheless, ESMO is the most liberal one. Our results can be used as guide for updates of the ABAMs (e.g., used statistical quantity and/or thresholds). Furthermore, using the current methods, we were able to calculate cutoff values for ASCO that correspond to ESMO and IQWiG categories, which improves the practical comparison between the methods after their application.

## Supplemental Material

sj-pdf-1-mdm-10.1177_0272989X241239928 – Supplemental material for A Comparison of Additional Benefit Assessment Methods for Time-to-Event Endpoints Using Hazard Ratio Point Estimates or Confidence Interval Limits by Means of a Simulation Study Supplemental material, sj-pdf-1-mdm-10.1177_0272989X241239928 for A Comparison of Additional Benefit Assessment Methods for Time-to-Event Endpoints Using Hazard Ratio Point Estimates or Confidence Interval Limits by Means of a Simulation Study by Christopher A. Büsch, Marietta Kirchner, Rouven Behnisch and Meinhard Kieser in Medical Decision Making
