# Supplementary material for: A Comparison of Additional Benefit Assessment Methods for Time-to-Event Endpoints Using Hazard Ratio Point Estimates or Confidence Interval Limits by Means of a Simulation Study
Source: Med Decis Making. 2024 May 9;44(4):365–79. doi: 10.1177/0272989X241239928 (PMC11102642; doi:10.1177/0272989X241239928)
Supplement: sj-pdf-1-mdm-10.1177_0272989X241239928 – Supplemental material for A Comparison of Additional Benefit Assessment Methods for Time-to-Event Endpoints Using Hazard Ratio Point Estimates or Confidence Interval Limits by Means of a Simulation Study [file sj-pdf-1-mdm-10.1177_0272989X241239928.pdf]

A comparison of additional benefit assessment methods for time-to-event endpoints using hazard ratio point estimate or confidence interval limits by means of a simulation study

Appendix

ADEMP-Structure

Morris et al.[1] proposed an ADEMP structure for planning a simulation study with the goal of improving the design, analysis, and report of simulations. The abbreviation "ADEMP" stands for Aims, Data-generating mechanisms, Estimands, Methods, and Performance measures and are described in detail below. Additional information of the results of the performed simulation study and the corresponding R-Code is available online:
github.com/cbuesch/ASCOvsIQWiGvsESMO.

Aim:
This simulation study aims at comparing the statistical aspects of the additional benefit assessment methods of ASCO, IQWiG_{RR} and ESMO in an overall survival setting. As there are two versions of IQWiG method considered, four methods are compared in total denoted by ASCO, IQWiG_{RR}, Mod-IQWiG_{HR}, and ESMO.
Especially, the question which statistical approach (point estimate, upper CI limit and lower CI limit) might be better for determination of additional benefit is of particular interest. In addition, we aim at determining which ASCO cutoff values consistent with categories of ESMO, IQWiG_{RR} and Mod-IQWiG_{HR}. Further information about all methods is provided in the corresponding publications[2, 3, 4, 5, 6].

Data-generating mechanisms:
We restrict the application to the case of a single phase III trial and do not consider the case where two or more phase III trials are used for a more precise estimation of parameters. Failure time, censoring time, sample size calculation, and the number of iterations for each trial scenario had to be determined / calculated to simulate realistic phase III trials. In the following, the data generation mechanism of the simulations is summarized including the required parameters and choice of distributions.
The following notation will be used: Let T be the event time, C the censoring time and A the accrual time, which are all assumed to be independent. The density, distribution, and survival functions are denoted with $f_.$, $F_.$ and $S_.$, respectively. Furthermore, dur denotes the duration of the study.

- Data generation for each trial: To simulate time-to-event data of a two-arm randomized clinical phase III trial, the following algorithm was used:

  1. Set seed.
  2. Generate independent failure times T with the failure times f_S and f_T for the control and treatment group, n_S and n_T times, respectively.

3. Generate independent right-censoring time C and independent administrative censoring time A, which takes the accrual time into account, for each patient and select the minimum out of C, A and T for the final observed time-to-event data.

Therefore, the final data tuple is of the form $(\min(T, C, A), \mathbb{1}(T \leq \min(C, A)))$, where the first and second entry represent the event time and cause of event (failure or censoring), respectively.

- Seeds of the simulations: To achieve comparability between the different scenarios, the same integer numbers were used as seeds at the beginning of the 10,000 iterations in each scenario. Therefore, 10,000 integer numbers were once randomly and without replacement drawn out of a sample ranging from 1 to 1 billion.

- Failure time distribution of control and treatment group (f_C and f_T):

  - In case of exponentially distributed failure times, the median overall survival time of the control group (med_C), designHR, and trueHR (designHR $\cdot$ HR$_{\text{var}}$) were fixed to a specific value to calculate the required λ_C and λ_T of the exponential distribution:

$$f_C \sim \exp(\lambda_C), \qquad f_T \sim \exp(\lambda_T)$$

    1. λ_C was calculated using the assumed med_C:

$$\text{med}_C = \frac{\ln(2)}{\lambda_C} \Rightarrow \lambda_C = \frac{\ln(2)}{\text{med}_C}$$

    2. λ_T was calculated using the trueHR and the proportional hazards assumption:

$$\text{trueHR} = \frac{h_T(t)}{h_C(t)} = \frac{\lambda_T}{\lambda_C} \overset{1.}{\Rightarrow} \lambda_T = \text{trueHR} \cdot \frac{\ln(2)}{\text{med}_C},$$

    where at "1." the calculated conversion of λ_C was used.

    The parameter HR$_{\text{var}}$ is needed to illustrate scenarios with incorrect assumed treatment effects for sample size calculation, i.e. designHR $\neq$ trueHR.

  - In case of Weibull distributed failure times, med_C, designHR, and trueHR were fixed to a specific value to calculate the required parameters:

$$f_C \sim \text{Weibull}(\lambda_C, k_C),$$

$$f_T \sim \text{Weibull}(\lambda_T, k_T)$$

    1. λ_C was calculated using the assumed med_C:

$$\text{med}_C = \frac{(\ln(2))^{1/k_C}}{\lambda_C} \Rightarrow \lambda_C = \frac{(\ln(2))^{1/k_C}}{\text{med}_C}$$

    2. λ_T was calculated using the trueHR and the proportional hazards assumption:

$$\text{trueHR} = \frac{h_T(t)}{h_C(t)} = \frac{\lambda_T^{k_T} \cdot k_T \cdot t^{k_T-1}}{\lambda_C^{k_C} \cdot k_C \cdot t^{k_C-1}} \overset{(*)}{=} \frac{\lambda_T^k}{\lambda_C^k}$$

$$\overset{1.}{\Rightarrow} \lambda_T = \left(\frac{\text{trueHR} \cdot \ln(2)}{\text{med}_C^k}\right)^{\frac{1}{k}},$$

where at "1." the calculated conversion of λ_C was used and at $(*)$ the shape parameter k was chosen to be identical for both treatment groups to achieve a constant hazard ratio over time ($k_C = k_T$). The parameter HR_{var} was needed to illustrate scenarios with incorrect assumed treatment effects for the sample size calculation, i.e. designHR $\neq$ trueHR.

– In case of <u>Gompertz</u> distributed failure times, med_C, designHR, and trueHR were fixed to a specific value to calculate the required parameters:

$$f_C \sim \text{gompertz}(a_C, b_C),$$

$$f_T \sim \text{gompertz}(a_T, b_T)$$

1. b_C was calculated using med_C:

$$\text{med}_C = \frac{1}{a_C} \cdot \ln\left(1 + \frac{a_C}{b_C} \cdot \ln(2)\right) \Rightarrow b_C = \frac{a_C \cdot \ln(2)}{\exp(\text{med}_C \cdot a_C) - 1}$$

2. b_T was calculated using the trueHR and the proportional hazards assumption:

$$\text{trueHR} = \frac{h_T(t)}{h_C(t)} = \frac{b_T \cdot \exp(a_T \cdot x)}{b_C \cdot \exp(a_C \cdot x)} \overset{(*)}{=} \frac{b_T}{b_C}$$

$$\overset{1.}{\Rightarrow} b_T = \frac{\text{trueHR} \cdot a \cdot \ln(2)}{\exp(\text{med}_C \cdot a) - 1},$$

where at "1." the calculated conversion of λ_C was used and at $(*)$ the shape parameter a was chosen to be same for both treatment groups to achieve a constant hazard ratio over time ($a_C = a_T$). The parameter HR_{var} was needed to illustrate scenarios with incorrect assumed treatment effects for the sample size calculation, i.e. designHR $\neq$ trueHR.

– In the case of <u>piece-wise exponentially</u> distributed failure times with an additional late treatment effect for the treatment group, med_C, designHR, and trueHR were fixed to a specific value. To achieve a late treatment effect for the treatment group, a piece-wise exponential distribution was chosen:

$$\text{F}_C(x) = 1 - \exp(-\lambda_C \cdot x),$$

$$\text{F}_T(x) = \begin{cases} 1 - \exp(-\lambda_C \cdot x) & , x \in [0, \text{start}_T] \\ 1 - \exp(-\lambda_C \cdot \text{start}_T) \cdot \exp(-\lambda_T \cdot (x - \text{start}_T)) & \text{otherwise,} \end{cases}$$

where $\text{F}_C(x)$ and $\text{F}_T(x)$ are the cumulative distribution functions of the treatment and control group, $\lambda_C > 0$ and $\lambda_T > 0$ are the parameters of the corresponding exponential distributions, and start_T ($= \frac{1}{3} \cdot \text{med}_C$) is the time point where the treatment effect sets in. The failure times of the treatment groups were generated using the inversion method by Kolonko[7, chapter 8]:
Assuming an uniform random variable U on the interval [0,1], $X := \text{F}_T^{-1}(U)$ is

F_T distributed, meaning $\mathbb{P}(X \leq t) = \mathrm{F}_T, t \in \mathbb{R}$. Therefore, the inversion of the cumulative distribution $\mathrm{F}_T(x)$ is given by

$$\mathrm{F}_T^{-1}(y) = \begin{cases} \frac{\ln(1-y)}{-\lambda_C} & , y \in [0, 1 - \exp(-\lambda_C \cdot \text{start}_T)] \\ \frac{\ln(1-y)+\lambda_C \cdot \text{start}_T}{-\lambda_T} + \text{start}_T & \text{otherwise.} \end{cases}$$

Additionally, λ_C and λ_T were defined in the same way as in the standard exponential case.

- Censoring time distribution: To simulate a realistic phase III study, independent administrative censoring and independent right-censoring was generated. We achieved the targeted censoring proportion p_C in all scenarios without biasing the HR estimation; hence administrative censoring, independent right-censoring and failure times are independent from each other:

  1. *Censoring proportion for administrative censoring with an accrual period:*
     A patient with event time $T = t$, who enters the trial at time point $A = a$ after study initiation, will be censored if the event would happen after end of the study, i.e. if $t + a > dur$.

$$\begin{aligned} p_C &= \mathbb{P}[T + A > dur] \\ &= \int \mathbb{P}[T + A > dur \mid A = a] f_A(a) da \\ &= \int \mathbb{P}[T > dur - a] f_A(a) da \\ &= \int S_T(dur - a) f_A(a) da \end{aligned}$$

  2. *Censoring proportion for administrative and independent censoring:*
     In this case it is assumed that all patients are recruited at the same time; hence a patient can be censored for two reasons: Firstly, administratively if both his censoring time $C = c$ and his event time $T = t$ are later than the study duration ($dur < \min(c, t)$), meaning that the observed patient did not have an event and could be followed-up (e.g. did not move away) over the whole observation period. Secondly, independently if censoring occurs before the event and the study duration ($c < \min(t, dur)$), e.g. a patient moves away and cannot be followed-up anymore). It is obvious that a patient whose event time is after the end of the study ($T > dur$) must be censored. This can either happen administratively at the last follow-up visit or because a censoring event happened during the course of the study. Patients whose event would occur during the study ($T \leq dur$) will only be censored if the censoring event happens before this event. Taking this into account, the censoring proportion can be calculated solving the following integral:

$$\begin{aligned} p_C &= \int_0^{dur} \int_0^t f_{T,C}(t,c) dc dt + \int_{dur}^{\infty} \underbrace{\int_0^{\infty} f_{T,C}(t,c) dc \, dt}_{f_T(t)} \\ &= \int_0^{dur} f_T(t) F_C(t) dt + S_T(dur) \end{aligned}$$

3. *Censoring proportion for administrative (with accrual period) and independent censoring:*
The two above explained considerations need to be combined. This means that the time under observation for each patient reduces by the time $A = a$ that the patient enters the study after its initiation. Hence, integrating the expression from the last step over the accrual distribution yields:

$$\begin{aligned} p_C &= \int \left(\int_0^{dur-a} f_T(t)F_C(t)dt + S_T(dur-a) \right) f_A(a)da \\ &= \int \int_0^{dur-a} f_T(t)F_C(t)f_A(a)dtda + \int S_T(dur-a)f_A(a)da. \end{aligned}$$

Below, examples of the p_C calculations for exponential and Weibull failure time distributions are illustrated:
The event time T in a phase III clinical trial consists out of the control event time T_1 and treatment event time T_2, which have both the same underlying distribution. Nevertheless, to simulate a trial with a present positive treatment effect, the parameters of the underlying distributions of T_1 and T_2 are different, resulting in a hyper distributed failure time distribution $T = p_1 \cdot T_1 + p_2 \cdot T_2$, where p_1 and p_2 are the probabilities of control end treatment group, respectively.

Assuming hyper-exponential distributed failure time T:
In case of a hyper-exponential failure time distribution with $T_1 \sim \text{Exp}(\lambda_1)$ and $T_2 \sim \text{Exp}(\lambda_2)$, the density, distribution and survival functions are defined the following way:

$$\begin{aligned} F_T(t) &= p_1 \cdot (1 - \exp(-\lambda_1 \cdot t)) + p_2 \cdot (1 - \exp(-\lambda_2 \cdot t)) \\ f_T(t) &= p_1 \cdot (\lambda_1 \cdot \exp(-\lambda_1 \cdot t)) + p_2 \cdot (\lambda_2 \cdot \exp(-\lambda_2 \cdot t)) \\ S_T(t) &= p_1 \cdot \exp(-\lambda_1 \cdot t) + p_2 \exp(-\lambda_2 \cdot t) \end{aligned}$$

Assuming additionally independent exponential censoring time $C \sim \text{Exp}(\lambda_C)$ and uniform accrual time $A \sim \text{Unif}[0, a_{max}]$, the censoring probability p_C can be calculated using equation of step 3:

$$\begin{aligned} p_C &= \int_0^{a_{max}} \int_0^{dur-a} f_T(t)F_C(t)f_A(a)dtda + \int_0^{a_{max}} S_T(dur-a)f_A(a)da \\ &= \int_0^{a_{max}} \int_0^{dur-a} (p_1 \cdot (\lambda_1 \cdot \exp(-\lambda_1 \cdot t)) + p_2 \cdot (\lambda_2 \cdot \exp(-\lambda_2 \cdot t))) \cdot (1 - \exp(-\lambda_C \cdot t)) \cdot \frac{1}{a_{max}} dtda + \\ &\int_0^{a_{max}} (p_1 \cdot \exp(-\lambda_1 \cdot (dur-a)) + p_2 \exp(-\lambda_2 \cdot (dur-a))) \cdot \frac{1}{a_{max}} da \\ &= \frac{1}{a_{max}} \Bigg(\frac{p_1\lambda_1}{(\lambda_1+\lambda_C)^2} \Big(\exp((a_{max}-dur)(\lambda_1+\lambda_C)) - \exp(-dur(\lambda_1+\lambda_C)) \Big) + \\ &\quad \frac{p_2\lambda_2}{(\lambda_2+\lambda_C)^2} \Big(\exp((a_{max}-dur)(\lambda_2+\lambda_C)) - \exp(-dur(\lambda_2+\lambda_C)) \Big) \Bigg) + \\ &\quad p_1 + p_2 - \frac{p_1\lambda_1}{\lambda_1+\lambda_C} - \frac{p_2\lambda_2}{\lambda_2+\lambda_C} \end{aligned}$$

To achieve the targeted censoring proportion p_C, this equation needs to be solved for λ_C. Since no numerical solution is available, a numerical approximation algorithm for minimization without derivatives introduced by Brent[8], and implemented in the uniroot function of the stats package in R, was used instead.

Assuming hyperweibull distributed failure time T:
In case of a hyperweibull failure time distribution with $T_1 \sim \text{Weib}(\lambda_1, k_1)$ and $T_2 \sim \text{Weib}(\lambda_2, k_2)$, the density, distribution and survival functions are defined the following way:

$$
\begin{aligned}
F_T(t) &= p_1 \cdot \left(1 - \exp(-(t\lambda_1)^{k_1})\right) + p_2 \cdot \left(1 - \exp(-(t\lambda_2)^{k_2})\right) \\
f_T(t) &= p_1 \cdot \left((k_1\lambda_1 \cdot (t\lambda_1)^{k_1-1} \exp(-(t\lambda_1)^{k_1}))\right) + p_2 \cdot \left((k_2\lambda_2 \cdot (t\lambda_2)^{k_2-1} \exp(-(t\lambda_2)^{k_2}))\right) \\
S_T(t) &= p_1 \cdot \exp(-(t\lambda_1)^{k_1}) + p_2 \cdot \exp(-(t\lambda_2)^{k_2})
\end{aligned}
$$

Assuming additionally independent exponential censoring time $C \sim \text{Exp}(\lambda_C)$ and uniform accrual time $A \sim \text{Unif}[0, a_{max}]$, the censoring probability p_C can be calculated using equation of step 3:

$$
\begin{aligned}
p_C = &\int_0^{a_{max}} \int_0^{dur-a} f_T(t)F_C(t)f_A(a)dtda + \int_0^{a_{max}} S_T(dur-a)f_A(a)da \\
= &\int_0^{a_{max}} \int_0^{dur-a} \left(p_1 \cdot \left(1 - \exp(-(t\lambda_1)^{k_1})\right) + p_2 \cdot \left(1 - \exp(-(t\lambda_2)^{k_2})\right)\right) \cdot \\
&(1 - \exp(-\lambda_C t)) \cdot \frac{1}{a_{max}} dtda + \\
&\int_0^{a_{max}} \left(p_1 \cdot \exp(-((dur-a)\lambda_1)^{k_1}) + p_2 \cdot \exp(-((dur-a)\lambda_2)^{k_2})\right) \cdot \frac{1}{a_{max}} da \\
= &\, p_1 + p_2 - \frac{1}{a_{max}} \left(\int_0^{a_{max}} \int_0^{dur-a} p_1 k_1 \lambda_1 (t\lambda_1)^{k_1-1} \exp(-(t\lambda_1)^{k_1}) \exp(-\lambda_C t) + \right. \\
&\left. p_2 k_2 \lambda_2 (t\lambda_2)^{k_2-1} \exp(-(t\lambda_2)^{k_2}) \exp(-\lambda_C t) dtda \right)
\end{aligned}
$$

Unfortunately, the remaining double integral cannot be solved analytically. Furthermore, to achieve the targeted censoring proportion p_C, this equation needs to be solved for λ_C. Thus, the remaining double integral and solving for λ_C was achieved using numerical approximation. Numerical approximation for solving the double integral was performed by an algorithm proposed by Piessens[9], which is implemented in the integrate function of the stats package in R. Furthermore, numerical approximations for solving for λ_C was performed by an algorithm for minimization without derivatives introduced by Brent[8], which is implemented in the uniroot function of the stats package in R.

- <u>Sample size calculations</u> were performed for each sub-scenario with the approach of Schoenfeld [10, 11]:

  1. Calculate the required number of events:

  $$d = \frac{(1+r)^2}{r} \cdot \frac{(\mathbf{z}_{1-\frac{\alpha}{2}} + \mathbf{z}_{1-\beta})^2}{(\ln(\text{designHR}))^2},$$

     where α is the type-I-error rate, β is the type-II-error rate, r is the sample size ratio between the treatment and the control group ($r = n_T/n_C$), designHR is the expected hazard ratio / treatment effect, and $\mathbf{z}_{1-\frac{\alpha}{2}}$ as well as $\mathbf{z}_{1-\beta}$ are the standard normal percentiles.

  2. Calculate the probability of an event $P(D)$ and divide the number of required events d by this probability to get the required sample size N. Hence, $N = \frac{d}{P(D)} = \frac{d}{p_C}$.

- <u>Scenarios / specification of parameters</u>
  An extensive simulation study was performed to provide the aspired detailed overview between the two methods by generating different scenarios of phase III trials:

  1. *Standard Scenario (Scenario 1):* Exponentially distributed failure times using
     - $\text{med}_C \in \{6, 12, 18, 24, 30\}$
     - designHR $\in \{0.3, 0.32, 0.34, ..., 0.86, 0.88, 0.9\}$
     - $\text{HR}_{\text{var}} = 1$
     - $\beta \in \{0.1, 0.2\}$
     - $\alpha = 0.05$ (two-sided)
     - $r = 1$
     - Combination out of administrative censoring (accrual time of 2 years and a follow-up time of $2 \cdot \text{med}_C$) and exponential censoring so that a censoring rate of 60% ($p_C = 0.6$) was achieved.

  2. *Incorrect assumed treatment effect (Scenario 2):* To achieve over- and under-powered studies, the same parameters were used as in Scenario 1, except designHR$\neq$ trueHR was chosen: $\text{HR}_{\text{var}} \in \{0.8, 0.9, 1.1, 1.2\}$.

  3. *Two different parameter distributions (Scenario 3):* Weibull and Gompertz distributions were used instead of exponential distribution as failure time distributions. To achieve proportional hazards for Weibull and Gompertz distributions, the shape parameter of each distribution was fixed to two different values, causing the hazard function to increase/decrease over time. An example using a designHR of 0.9 (designHR=trueHR) and med_C of 6 months can be found in Appendix Figure 1.
     With Weibull (and Gompertz) distributed failure times and shape parameter smaller than 1 (smaller than 0), the hazard rate is a monotonically decreasing function and thus the hazard of an event is getting smaller over time, which means that if a patient survives until a certain time t, he/she has a high probability of surviving until end of study. Hence, these patients are getting censored at the end of the study (administrative censoring). Since, these distributions were specifically chosen to simulate increasing and decreasing hazards (and not constant hazards like exponential distribution) and to have a censoring mechanism which achieves the targeted censoring proportions without biasing the hazard ratio estimations, a 60% censoring rate was targeted. Furthermore, to still be able to have comparable scenarios,

independent exponential censoring was added with the goal to have similar overall censoring rates in all scenarios; e.g. for scenarios with administrative censoring rates of <60% independent exponential censoring was added so that an overall censoring of 60% was achieved leading to comparable scenarios.
Unfortunately, even with a targeted censoring rate of 60% some sub-scenarios of Scenario 3b (Gompertz) resulted in an administrative censoring rate larger than the targeted 60%. To still have comparable scenarios, we excluded these sub-scenarios of Scenario 3b leading to 1184 sub-scenarios instead of 1240.

4. *Delayed treatment effect (Scenario 4):* Delayed treatment effect for the treatment group, which is a kind of non-proportional hazards, using piece-wise exponential failure time distributions. For this objective, the same parameters as in the Standard Scenario were used. The underlying distribution of the treatment group, $F_T(x)$, was chosen to be exponential using the distribution parameter λ_C of the control group until start_T and λ_T after start_T (delayed treatment effect):

$$F_C(x) = 1 - \exp(-\lambda_C \cdot x)$$

$$F_T(x) = \begin{cases} 1 - \exp(-\lambda_C x) & , x \in [0, \text{start}_T] \\ 1 - \exp(-\lambda_C \cdot \text{start}_T) \cdot \exp(-\lambda_T \cdot (x - \text{start}_T)) & , \text{otherwise} \end{cases}$$

where F_C and F_T are the cumulative distribution functions of the treatment and control group, $\lambda_C > 0$ and $\lambda_T > 0$ are the parameters of the corresponding exponential distributions, and start_T ($= \frac{1}{3} \cdot \text{med}_C$) is the time point of treatment effect start for the treatment group. The failure times of the treatment groups were generated using the inversion method by Kolonko [7, chapter 8]. Hence proportional hazards were assumed before and after start_T. Additionally, λ_C and λ_T were defined the same way as in the Standard Scenario.
ESMO uses the gain of the new treatment (absolute difference of median treatment outcomes) to establish the different categories. Hence, if $\text{med}_T \approx \text{med}_C$, the method would only assign the lowest score to a new treatment. To not penalize ESMO for its design, start_T was set to $\frac{1}{3}$ of the assumed median survival time of the control group (med_C) for the simulations ($\text{start}_T \ll \text{med}_C$). An example using a designHR of 0.7 (designHR=trueHR), med_C of 12 months and start_T of 4 months can be found in Appendix Figure 2.

- Software: The simulation was performed using the software R version 4.2.1, with packages "tidyverse", “survival”, “flexsurv”, "cutpointr", "vcd" and "pcaPP" for data generation and analysis. Additionally, the package "ggpubr" was used for illustrations.

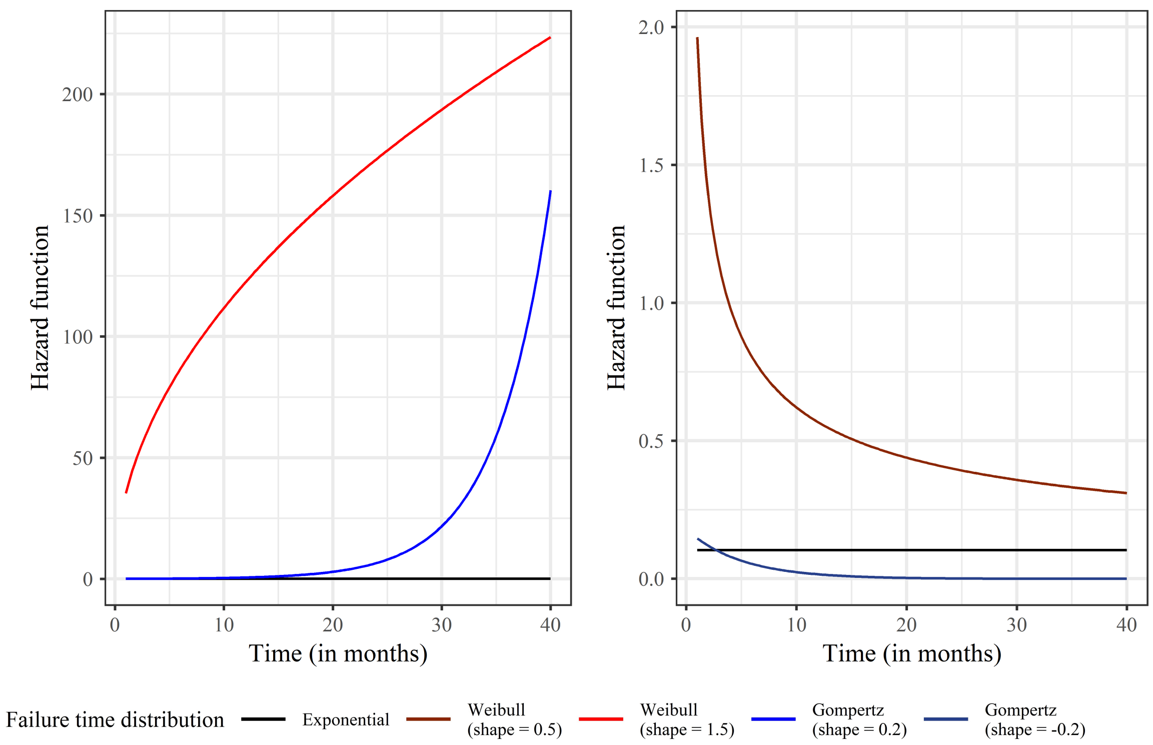

Appendix Figure 1: Hazard functions of exponential, Weibull, and Gompertz distribution with assumed parameters for a designHR = 0.9, designHR=trueHR and $\text{med}_C = 6$ months

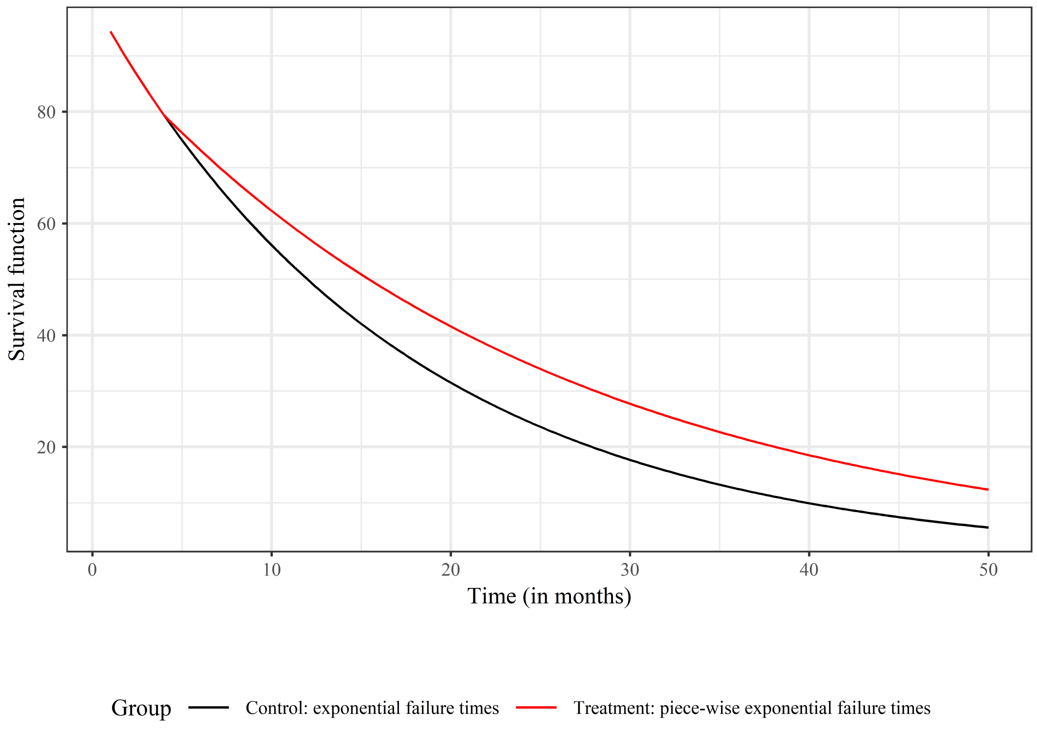

Appendix Figure 2: Survival functions of piece-wise exponential distribution with late treatment effect, assuming a designHR of 0.7, designHR=trueHR, $\text{med}_C = 12$ months and $\text{start}_T = 4$ months ($\text{start}_T = \frac{1}{3} \cdot \text{med}_C$).

Estimands:

Since in this simulation study the aim is not a simple parameter of the data generating model, we are more interested in different targets:

1. Estimation of the relationship between the methods using pairwise correlation, $\theta_1(x, y)$, where x and y are two of the four methods (ASCO, IQWiG$_{\text{RR}}$, Mod-IQWiG$_{\text{HR}}$, and ESMO).

2. Estimation of median, θ_2^{ASCO}, for method with continuous outcome (ASCO) and scoring category rates for methods with ordinal outcome, $\theta_2^{i,j}$, where i and j represent category i of method j=ASCO, IQWiG$_{\text{RR}}$, Mod-IQWiG$_{\text{HR}}$, and ESMO, respectively.

3. Estimation of ASCO cutoff values, which are consistent with categories of ESMO, IQWiG$_{\text{RR}}$ and Mod-IQWiG$_{\text{HR}}$.

Methods:

Below, the benefit assessment methods are presented. As there are two version of IQWiG method considered, four methods are compared in total denoted by ASCO, IQWiG$_{\text{RR}}$, Mod-IQWiG$_{\text{HR}}$, and ESMO. The methods are applied for a statistically significant phase III trial based on the log-rank test. We restrict the application to single phase III trials and do not consider cases where two or more phase III trials are used for more precise parameter estimations. In an attempt to achieve a fair comparison for the statistical aspects of the method in an overall survival / advanced diseases framework, only the clinical benefit score and tail of the curve bonus points of NHB score of ASCO was carried out. Furthermore, ESMO score 5 was not used because it can only be achieved with additional bonus points adjustments, e.g. toxicity improvements. Hence, ESMO ranges from 1 until 4. Below the four additional benefit assessment methods used in our simulation study are described. Further information on how the methods are calculated is provided in the corresponding publications[2, 3, 4, 5, 6].

1. ASCO uses a sum of a clinical benefit score, toxicity score, and bonus points to calculate the NHB score. As main component the NHB defines the clinical benefit score, which uses the HR-PE to calculate a continuous value: $100 \cdot (1 - \text{HR-PE})$. The toxicity score is limited to 20 points and defined as the percentage differences in total toxicity points between the two treatment arms, which is based on the graded toxicities between 0.5 and 2.0 in each treatment arm. The bonus points consist of many different aspects including palliation (cancer related symptoms), quality of life, treatment-free interval bonus and the tail of the survival curve. For the latter, the time point on the survival curve that is two times the median OS of the control arm ($2 \cdot \text{med}_{\text{C}}$) is identified. If the proportion of patients alive in the treatment compared to the control arm improved by 50% or greater (assuming > 20% surviving in control arm), 20 points are rewarded. Table1 gives an overview of ASCO.
   Important: In the simulation study only the statistical parts of the method is used. Hence, only clinical benefit score and the bonus point tail of the curve are implemented.

2. IQWiG$_{\text{RR}}$ evaluates the additional benefit of new drugs using the upper limit of the HR-CI (HR^+) and bonus point adjustments grading drugs into three categories (major, considerable, and minor added benefit) reflecting not only on HR^+ but also on toxicity, quality of life and other important endpoints. For the main classification, which isused here,

the HR^+ estimates are compared to Relative Risk (RR) scaled thresholds 0.85 and 0.95. Thus $HR^+<0.85$ is considered as major, $0.85< HR^+<0.95$ as considerable and $HR^+>0.95$ as minor added benefit. Table1 gives an overview of $IQWiG_{RR}$.
Important: In the simulation study only the statistical parts of the method is used. Hence, no bonus point adjustments are implemented.

3. In addition, as proposed by Büsch et.al[12], we transformed the RR-scaled $IQWiG_{RR}$ thresholds with VanderWeele conversion formula[13] into HR-scaled thresholds (Mod-$IQWiG_{HR}$).

$$\mathrm{RR} = \frac{1 - 0.5^{\sqrt{\mathrm{HR}}}}{1 - 0.5^{\sqrt{\frac{1}{\mathrm{HR}}}}}$$

Since this formula has no analytical solution for HR, we used a numerical approach to calculate the HR-scaled thresholds, leading to the thresholds 0.79 and 0.93. Table1 gives an overview of $IQWiG_{RR}$.

4. ESMO has developed a combination of relative benefit using the lower limit of the 95% HR-CI (HR^-), absolute benefit using the gain definition, and bonus point adjustments. These estimates are compared to specific thresholds leading to ordinal rating for the classification, comprising out of five categories, where grades 5 and 4 represent substantial and grades 3 to 1 low benefit; for example a trial with a median survival time in the control group (med_C) between 12 and 24 months and the $HR^-<0.75$ or gain<1.5 months, would be awarded grade 1. Furthermore, grade 5 can only be achieved after bonus point adjustments, reflecting on toxicity and quality of life of the new treatment. Grade 4 can already be achieved if the survival rate increases by 10% or more at key milestones. For a more detailed overview of the construction and specific thresholds Table1 provides an overview of ESMO.
Important: In the simulation study only the statistical parts of the method is used. Hence, no bonus point adjustments are implemented.

For the subsequent application of the methods the HR-PE with corresponding 95% Wald-CI, and the 2-, 3- and 5-year survival increase were required. Additionally, for ASCO bonus point adjustment "tail of the curve" and ESMO absolute benefit rule med_C or med_T had to be calculated. However, if the survival curve does not fall below 50%, e.g. due to large treatment effects, the median survival time cannot be observed. As in Büsch et al.[12] a conservative approach was implemented, using the last observed censoring or event time point of the survival curve instead.

ASCO			
clinical benefit score (CB)	toxicity score (T)	bonus points (BP)	net health benefit score (NHB)
$\text{CB} = 100 \cdot (1 - \text{HR-PE})$	1. Calculate total toxicity points for each treatment arm:<br>Sum of each clinically meaningful toxicity, which is assigned a score between 0.5 and 2.0 based on grade and frequency.<br>2. Calculate the percentage differences, p_{Diff}, in total toxicity points between the two treatment arms.<br>3. $\text{T} = \begin{cases} 100 \cdot p_{\text{Diff}}, p_{\text{Diff}} \leq 0.2 \\ 20, \text{ otherwise} \end{cases}$	Many different aspects including palliation (cancer related symptoms), quality of life, treatment-free interval bonus and the tail of the survival curve. For the latter, the time point on the survival curve that is $2 \cdot \text{med}_{\text{C}}$, is identified. If the proportion of patients alive in the treatment compared to the control arm improved by 50% or greater (assuming > 20% surviving in control arm), 20 points are rewarded.	NHB = CB+T+BP

ESMO				
	1 (low benefit)	2 (low benefit)	3 (low benefit)	4 (substantial improvement)
$\text{med}_C \leq 12$	$\text{HR}^- > 0.7$<br>OR<br>gain < 1.5	[$\text{HR}^- \leq 0.65$ AND gain $\in [1.5, 2)$]<br>OR<br>[$\text{HR}^- \in (0.65, 0.7]$ AND gain ≥ 1.5]	$\text{HR}^- \leq 0.65$ AND gain $\in [2, 3)$	[$\text{HR}^- \leq 0.65$ AND gain ≥ 3]<br>OR<br>[Increase in 2 year survival $\geq 10\%$]
$\text{med}_C \in (12, 24]$	$\text{HR}^- > 0.75$<br>OR<br>gain < 1.5	[$\text{HR}^- \leq 0.7$ AND gain $\in [1.5, 3)$]<br>OR<br>[$\text{HR}^- \in (0.7, 0.75]$ AND gain ≥ 1.5]	$\text{HR}^- \leq 0.7$ AND gain $\in [3, 5)$	[$\text{HR}^- \leq 0.7$ AND gain ≥ 5]<br>OR<br>[Increase in 3 year survival $\geq 10\%$]
$\text{med}_C > 24$	$\text{HR}^- > 0.75$<br>OR<br>gain < 4	[$\text{HR}^- \leq 0.7$ AND gain $\in [4, 6)$]<br>OR<br>[$\text{HR}^- \in (0.7, 0.75]$ AND gain ≥ 4]	$\text{HR}^- \leq 0.7$ AND gain $\in [6, 9)$	[$\text{HR}^- \leq 0.7$ AND gain ≥ 9]<br>OR<br>[Increase in 5 year survival $\geq 10\%$]

IQWiG_{RR}		
minor added benefit	considerable added benefit	major added benefit
$\text{HR}^+ \in [0.95, 1)_{\text{RR}}$	$\text{HR}^+ \in [0.85, 0.95)_{\text{RR}}$	$\text{HR}^+ < 0.85_{\text{RR}}$

$\text{Mod-IQWiG}_{\text{HR}}$		
minor added benefit	considerable added benefit	major added benefit
$\text{HR}^+ \in [0.93, 1)_{\text{HR}}$	$\text{HR}^+ \in [0.79, 0.93)_{\text{HR}}$	$\text{HR}^+ < 0.79_{\text{HR}}$

Table 1: Modified figure from Büsch et. al[12]: Categories of IQWiG_{RR}, $\text{Mod-IQWiG}_{\text{HR}}$ and ESMO for overall survival / advanced diseases framework. Additionally, the statistical parts of ASCO is presented.

Performance measures and n_{sim}:

Performance measures:

1. To estimate the relationship between the methods, pairwise Spearman correlation was calculated examining the complete range of categories for the two methods:

$$\widehat{\theta}_1(x, y) = \frac{\sum x_i y_i - n\bar{x}\bar{y}}{(n-1)s_x s_y},$$

   where n is the sample size, x_i, $\bar{x}$ and s_x are the individual scores, sample means and sample standard deviations of the rank-converted scores of the additional benefit assessment method x. Analogously, the same applies for y_i, $\bar{y}$ and s_y for additional benefit assessment method y.

2. To estimate the category rates of each methods (with an ordinal outcome) and each sub-scenario, the proportion is used:

$$\widehat{\theta}_2 = \frac{M_i^j}{\text{Number of significant trials}} = \frac{\text{Number of maximal scores}}{\text{Number of significant trials}},$$

   where M_i is the number of category i of method $j = \{$ASCO ESMO IQWiG_{RR}, Mod-$\text{IQWiG}_{\text{HR}}\}$. The amount of categories differ for each method:

   - IQWiG_{RR} and Mod-IQWiG_{HR}: "minor added benefit", "considerable added benefit" and "major added benefit"
   - ESMO: 1, 2, 3, 4

   Since ASCO has a continuous outcome, estimated median, θ_2^{ASCO}, is reported.

3. To investigate which ESMO, IQWiG_{RR} and Mod-IQWiG_{HR} category corresponds to which ASCO score, maximizing weighted Cohens kappa approach was used for cutoff value determination[14, 15]:

$$\kappa = 1 - \frac{\sum_{i=1}^{k}\sum_{j=1}^{k} w_{ij} \cdot x_{ij}}{\sum_{i=1}^{k}\sum_{j=1}^{k} w_{ij} \cdot m_{ij}},$$

   where $i = 1, \ldots, k$ and $j = 1, \ldots, k$ are the categories of both raters / methods, x is the observed probability matrix, w the quadratic weights matrix and m the expected probability matrix. Since ESMO and IQWiG_{RR} are ordinal scores, disagreements close to the diagonal implies a smaller disagreement than far from the diagonal. Thus, Cohen kappa with "Fleiss-Cohen" were used.
   As sensitivity analysis for optimal cutoff determination receiver operating characteristic (ROC) curves were used dividing categories pairwise, and considered optimal when the point on the ROC curve is closest to the point (0,1) (ROC01). As second sensitivity analysis Svenssons method[16, 17] was used, which defines cutoffs so that same marginal distribution of ordinal method and continuous ASCO are present.

Number of iterations for each scenario (n_{sim}):
As this performed simulation study was initially planned to assess the differences between IQWiG and ESMO, n_{sim} was defined the following way:
In each simulated scenario, it was sought to achieve a standard deviation of 0.25% for the coverage probability of a maximal ESMO grade or maximal IQWiG grade assuming constant error variance. For each iteration of a scenario, the confidence interval either covers the true value or it does not. Thus, an indicator variable was defined:

$$Y_i = \begin{cases} 1, & \text{if the maximal grade is given,} \\ 0, & \text{if it is not given.} \end{cases}$$

Therefore, Y_i is a Bernoulli variable and the coverage probability $\mathbb{E}(Y_i) = p$ can be estimated by the sample proportion. Since the variance of a Bernoulli variable is given by $p \cdot (1-p)$ and the simulations generated independent and identically distributed Bernoulli variables, the variance of the simulation-based estimate of p is $\frac{p \cdot (1-p)}{n_{sim}}$, where n_{sim} is the number of iterations. In fact, it can be shown that

$$\frac{p \cdot (1-p)}{n_{sim}} \leq \frac{1}{4 \cdot n_{sim}}.$$

Therefore, to achieve a variance less than some pre-specified threshold δ, n_{sim} can be calculated using

$$n_{sim} \geq \frac{1}{(4 \cdot \delta)}.$$

Setting the threshold δ to $2.5 \cdot 10^{-5}$, which corresponds to a standard deviation of 0.5%, results in a number of $n_{sim} = 10,000$ iterations for each scenario.

Additional results and interpretation of performed simulation study

- Reason for ESMO assigning large proportion of the maximal category:
  Appendix Figure5 below shows the proportion of maximal ESMO, $IQWiG_{RR}$ and $Mod\text{-}IQWiG_{HR}$ categories for all sub-scenarios of the Standard Scenario. It clearly depicts that $IQWiG_{RR}$ and $Mod\text{-}IQWiG_{HR}$ have a large proportion of maximal score in case of large treatment effects (e.g. around 75% in case of designHR=0.3). ESMO, however, classifies over a very large range of treatment effects (between roughly designHR 0.3 and 0.6) almost always 100% maximal categories. Hence, ESMO is clearly more liberal than $IQWiG_{RR}$ and $Mod\text{-}IQWiG_{HR}$. This difference can further be seen over all sub-scenarios and thus is independent of underlying med_C and power.
  Appendix Figure4 provides a further illustration for the proportion of maximal ESMO category due to the survival gain rule, dual rule or both rules (number of maximal ESMO categories due to survival gain rule (OR dual rule OR both) divided by number of maximal ESMO categories) of our simulations for the Standard Scenario, showing that most of the times a maximal ESMO category was assigned by its dual rule as well as its survival gain rule. Only in scenarios with small treatment effects (designHR close to 1) the reason that ESMO assigns a maximal category is solely due to its survival gain rule (green line at 100%). The number of assigned maximal scores in these sub-scenarios, however, is small. For example, in the sub-scenario with med_C=12, power of 90% and designHR=0.88 (bottom row, second panel from the left), ESMO assigns 105 trials a maximal category; in sub-scenario with med_C=24, power of 80% and designHR=0.9 (top row, second panel from the left) ESMO assigns 21 trials a maximal category. Overall, it is safe to say that in our simulation all parts of the ESMO contribute to the ESMO assessment.
  The reason why ESMO has such a large proportion of the maximal category depends primarily on the choice of thresholds used. Büsch et. al already pointed out that the current used thresholds of ESMO lead to a very poor false positive rate and thus leading to easily achievable maximal category[12]. This fact, however, is not the main purpose of this paper and is already described in other publications.

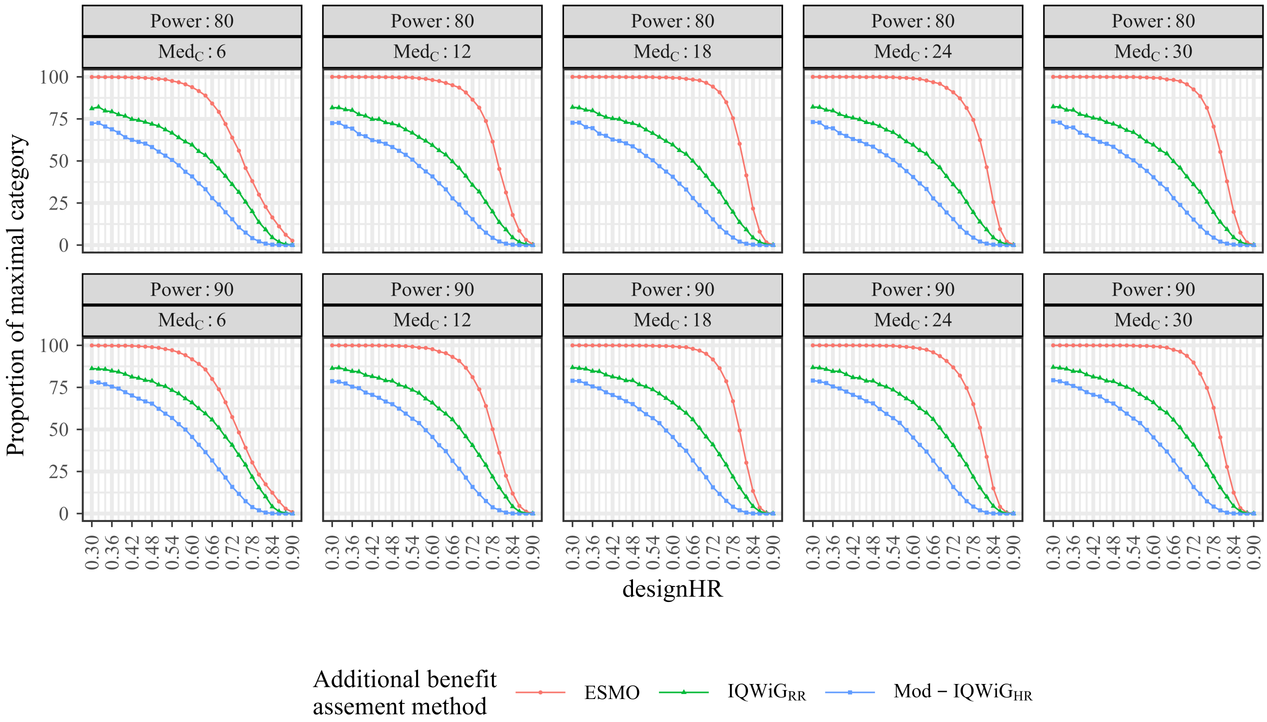

Appendix Figure 3: Proportion of maximal ESMO, IQWiG$_{RR}$ and Mod-IQWiG$_{HR}$ categories for all sub-scenarios of the Standard Scenario.

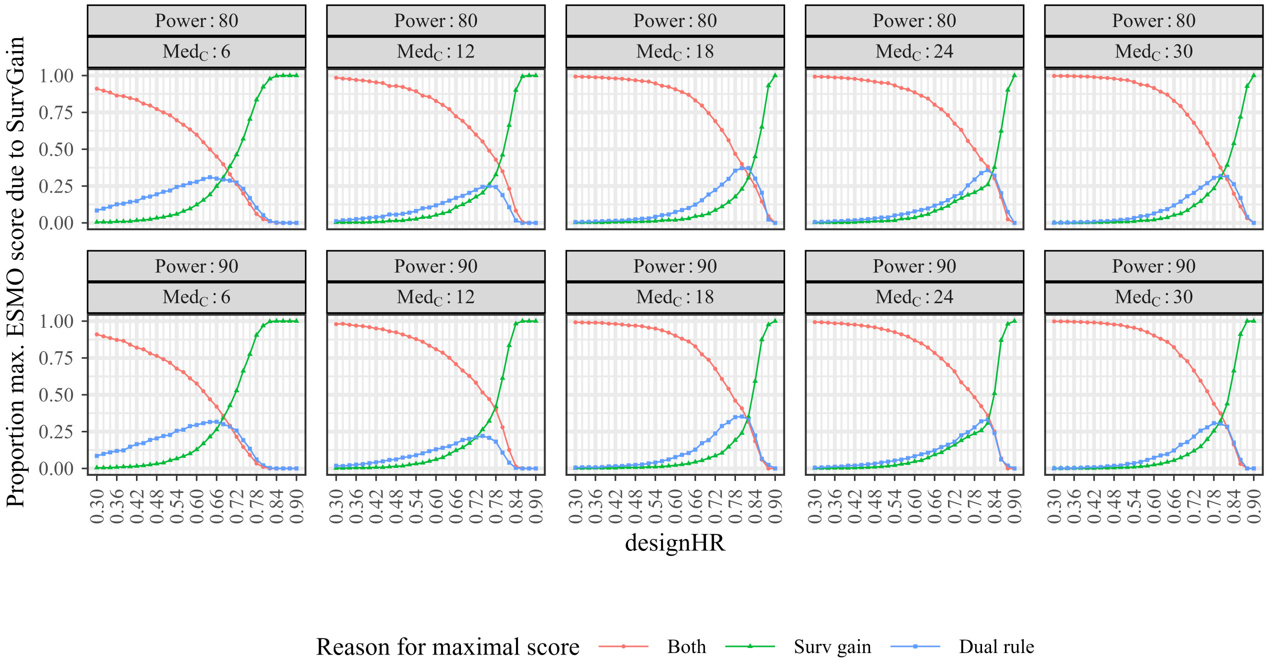

Appendix Figure 4: proportion of maximal ESMO category due to the survival gain rule, dual rule or both rules (number of maximal ESMO categories due to survival gain rule (OR dual rule OR both) divided by number of maximal ESMO categories).

- Kendall's τ_b as sensitivity analysis for Spearman correlation:
  It can be seen that the Kendall's τ_b results are consistent with the Spearman correlation. The only difference is that the Kendall's τ_b values are overall smaller than the Spearman correlation, which is often observed when comparing results of these two measurements.

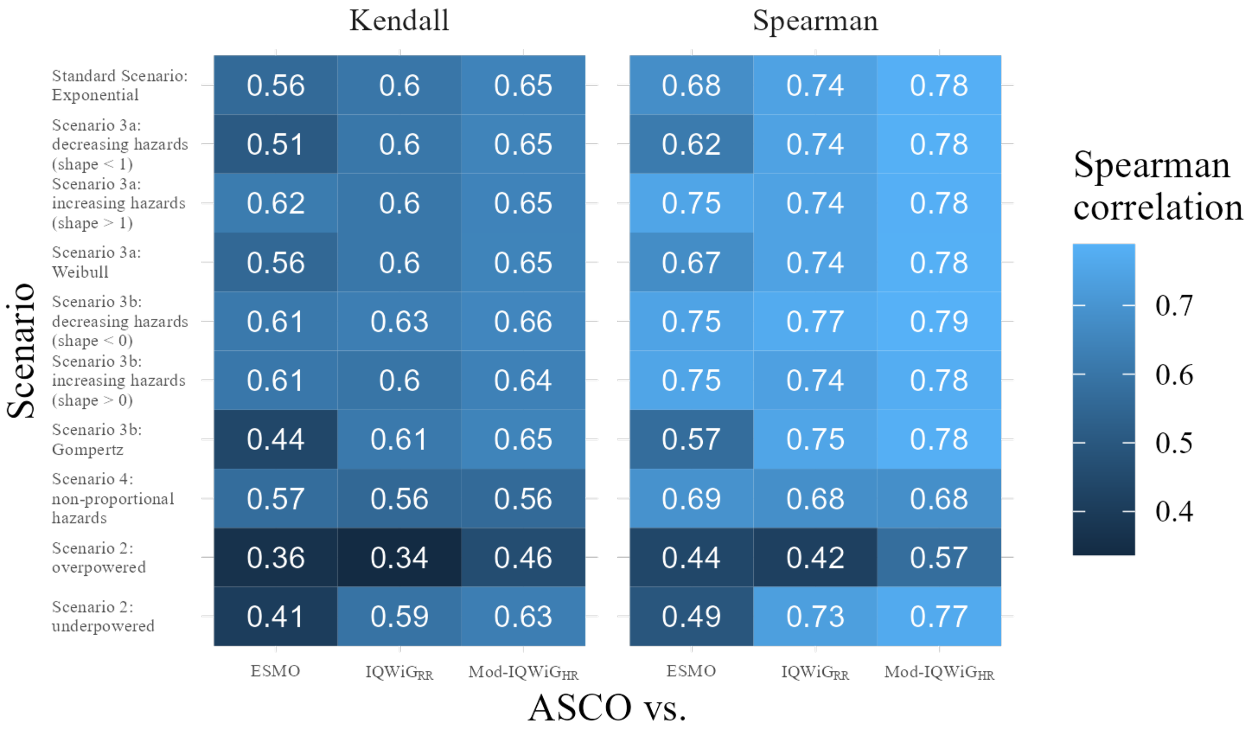

Appendix Figure 5: Pairwise Spearman correlation and Kendall's τ_b results between ASCO and ESMO / IQWiG$_{RR}$ / Mod-IQWiG$_{HR}$ method illustrated using heatmap for all scenarios with combined sub-scenarios, meaning the complete range of designHRs, HR$_{var}$, power, med$_C$ and shape was used together.

- Further description of Figure 5 and 6 of paper:
  Figure 5:
  Correlation between ASCO and Mod-IQWiG$_{HR}$ reaches its maximum at a designHR of 0.62 (for all medC values), instead of 0.72 for IQWiG$_{HR}$. This can be explained by the IQWiG$_{RR}$/Mod-IQWiG$_{HR}$ classification solely depending on the HR$^+$ estimate, which leads to the most uniform category distribution at a designHR of 0.72 or 0.62, respectively. For example, at small (large) designHRs almost every (no) study is assigned the maximal category, meaning that IQWiG$_{RR}$ category distribution is skewed. In case of moderate designHRs between 0.54 and 0.76, the majority of HR$^+$ estimates are between the thresholds 0.85 and 0.95 of IQWiG$_{RR}$ and hence the correlation between ASCO and IQWiG$_{RR}$ is the largest for these sub-scenarios.
  Furthermore, the correlation of both pairwise method comparisons decline after having reached the maximum before increasing again (Fig.5 of Paper), which has two causes: Firstly, only significant trials are used for the additional benefit assessment methods reducing the range of HR$^+$ estimates (maximal possible estimated HR$^+$ is 1). Secondly, the performed sample size calculation leads to larger sample sizes for sub-scenarios with a smaller treatment effect and thus to a smaller range of HR$^+$ estimates. This leads to less uniform distributed categories and hence reduced correlation. The again

increasing correlation at very small treatment effects (designHR>0.84) can be explained by hardly any maximal category assignments, leading to similar results. Furthermore, the remaining categories considerable and minor added benefit are more uniformly distributed with designHRs closer to 1, as the threshold of $IQWiG_{RR}$ nears 1 as well.

Figure 6:
Different underlying distributions only affect the correlation between ASCO and ESMO. Splitting sub-scenarios with Gompertz distribution in increasing (Scenario 3b, shape>0) and decreasing hazards (shape<0), however, leads to a constant correlation of 0.75. This can be explained by looking at the scoring description of both methods. With underlying decreasing hazards, ASCO awards a smaller score with median of 33 compared to increasing hazards of 41. Furthermore, with decreasing hazards, ESMO categories are very skewedly distributed; for example, only 0.03% of the trials were categorized into category 3 vs. 16.48% in case of increasing hazards. Combining these two observations shows that in Scenario 3a ASCO mainly originate from ESMO category 3 with increasing hazards, leading to decreasing ASCO with increasing ESMO category (Fig.2, top right panel), causing a decreased correlation.

References

[1] T. Morris, I. White, and M. Crowther, “Using simulation studies to evaluate statistical methods,” *Statistics in Medicine*, no. 38, pp. 2074–2102, 2019.

[2] L. E. Schnipper, N. E. Davidson, D. S. Wollins, C. Tyne, D. W. Blayney, D. Blum, A. P. Dicker, P. A. Ganz, J. R. Hoverman, R. Langdon, G. H. Lyman, N. J. Meropol, T. Mulvey, L. Newcomer, J. Peppercorn, B. Polite, D. Raghavan, G. Rossi, L. Saltz, D. Schrag, T. J. Smith, P. P. Yu, C. A. Hudis, and R. L. Schilsky, “American society of clinical oncology statement: A conceptual framework to assess the value of cancer treatment options,” *Journal of clinical oncology*, vol. 33, no. 23, pp. 2563–U132, 2015.

[3] L. E. Schnipper, N. E. Davidson, D. S. Wollins, D. W. Blayney, A. P. Dicker, P. A. Ganz, J. R. Hoverman, R. Langdon, G. H. Lyman, N. J. Meropol, T. Mulvey, L. Newcomer, J. Peppercorn, B. Polite, D. Raghavan, G. Rossi, L. Saltz, D. Schrag, T. J. Smith, P. P. Yu, C. A. Hudis, J. M. Vose, and R. L. Schilsky, “Updating the american society of clinical oncology value framework: Revisions and reflections in response to comments received,” *Journal of clinical oncology*, vol. 34, no. 24, pp. 2925–2934, 2016.

[4] G. Skipka, B. Wieseler, T. Kaiser, *et al.*, “Methodological approach to determine minor, considerable, and major treatment effects in the early benefit assessment of new drugs,” *Biometrical Journal*, no. 58, pp. 43–58, 2016.

[5] N. I. Cherny, R. Sullivan, U. Dafni, *et al.*, “A standardised, generic, validated approach to stratify the magnitude of clinical benefit that can be anticipated from anti-cancer therapies: the european society for medical oncology magnitude of clinical benefit scale (esmo-mcbs),” *Annals of Oncology*, no. 26, pp. 1547–1573, 2015.

[6] N. I. Cherny, U. Dafni, J. Bogaerts, *et al.*, “Esmo-magnitude of clinical benefit scale version 1.1,” *Annals of Oncology*, no. 28, pp. 2340–2366, 2017.

[7] M. Kolonko, *Stochastische Simulation: Grundlagen, Algoritmen und Anwendungen.* Springer: Vieweg+Teubner, GWV Fachverlage GmbH, Wiesbaden 2008, 1 ed., 2008.

[8] D. G. and R. P. Brent, “Algorithms for minimization without derivatives,” *Mathematics of Computation*, vol. 28, no. 127, pp. 865–866, 1974.

[9] R. Piessens, *QUADPACK.* No. ARRAY(0x563dcaa4b148) in Springer series in computational mathematics, Berlin ; Heidelberg [u.a.]: Springer, 1983. Literaturverz. S. 295 - 301.

[10] D. Schoenfeld, “The asymptotic properties of nonparametric tests for comparing survival distributions,” *Biometrika*, no. 68, pp. 316–319, 1981.

[11] D. Schoenfeld, “Sample-size formula for the proportional-hazards regression model,” *Biometrics*, no. 39, pp. 499–503, 1983.

[12] C. A. Büsch, J. Krisam, and M. Kieser, “A comprehensive comparison of additional benefit assessment methods applied by institute for quality and efficiency in health care and european society for medical oncology for time-to-event endpoints after significant phase iii trials—a simulation study,” *Value in health*, vol. 25, no. 11, pp. 1853–1862, 2022.

[13] T. J. VanderWeele, “Optimal approximate conversions of odds ratios and hazard ratios to risk ratios,” *Biometrics Biometric Methodology*, no. 76, pp. 746–752, 2020.

[14] J. Cohen, “A coefficient of agreement for nominal scales,” *Educational and psychological measurement*, vol. 20, no. 1, pp. 37–46, 1960.

[15] C.-H. Chang, J.-T. Yang, and M.-H. Lee, “A novel "maximizing kappa" approach for assessing the ability of a diagnostic marker and its optimal cutoff value,” *Journal of biopharmaceutical statistics*, vol. 25, no. 5, pp. 1005–1019, 2015.

[16] E. Svensson, “Comparison of the quality of assessments using continuous and discrete ordinal rating scales,” *Biometrical journal*, vol. 42, no. 4, pp. 417–434, 2000.

[17] E. Svensson, “Concordance between ratings using different scales for the same variable,” *Statistics in medicine*, vol. 19, no. 24, pp. 3483–3496, 2000.
